# Chemokines kill bacteria without triggering antimicrobial resistance by binding anionic phospholipids

**DOI:** 10.1126/sciadv.ads2675

**Published:** 2025-06-06

**Authors:** Sergio M. Pontejo, Sophia Martinez, Allison Zhao, Kevin Barnes, Jaime de Anda, Haleh Alimohamadi, Ernest Y. Lee, Acacia F. Dishman, Brian F. Volkman, Gerard C. L. Wong, David N. Garboczi, Angela Ballesteros, Philip M. Murphy

**Affiliations:** ^1^ Laboratory of Molecular Immunology, National Institute of Allergy and Infectious Diseases, National Institutes of Health, Bethesda, MD 20892, USA.; ^2^ Structural Biology Section, Research Technologies Branch, National Institute of Allergy and Infectious Diseases, National Institutes of Health, Bethesda, MD 20892, USA.; ^3^ Department of Bioengineering, University of California, Los Angeles, Los Angeles, CA 90095, USA.; ^4^ Department of Chemistry and Biochemistry, University of California, Los Angeles, Los Angeles, CA 90095, USA.; ^5^ Department of Microbiology, Immunology & Molecular Genetics, University of California Los Angeles, Los Angeles, CA 90095, USA.; ^6^ California NanoSystems Institute, University of California Los Angeles, Los Angeles, CA 90095, USA.; ^7^ Department of Biochemistry, Medical College of Wisconsin, Milwaukee, WI 53226, USA.; ^8^ Section on Sensory Physiology and Biophysics, National Institute on Deafness and Other Communication Disorders, National Institutes of Health, Bethesda, MD 20892, USA.

## Abstract

Classically, chemokines coordinate leukocyte trafficking; however, many chemokines also have direct antibacterial activity. The bacterial killing mechanism of chemokines and the biochemical properties that define which members of the chemokine superfamily are antimicrobial remain poorly understood. We report that the antimicrobial activity of chemokines is defined by their ability to bind phosphatidylglycerol and cardiolipin, two anionic phospholipids commonly found in the bacterial plasma membrane. We show that only chemokines able to bind these two phospholipids kill bacteria and that they exert rapid bacteriostatic and bactericidal effects with a higher potency than the antimicrobial peptide β-defensin 3. Both biochemical and genetic interference with the chemokine-cardiolipin interaction impaired microbial growth arrest, bacterial killing, and membrane disruption by chemokines. Moreover, unlike conventional antibiotics, *Escherichia coli* failed to develop resistance when placed under increasing antimicrobial chemokine pressure in vitro. Thus, we have identified cardiolipin and phosphatidylglycerol as binding partners for chemokines responsible for chemokine antimicrobial action.

## Teaser

The antimicrobial activity of chemokines is defined by their ability to bind anionic phospholipids.

## INTRODUCTION

The emergence of antimicrobial resistant bacteria, driven by widespread use of antibiotics and a decline in antibiotic innovation, is a major challenge to public health worldwide. Infections with antibiotic-resistant bacteria typically require prolonged hospital stays and cause higher morbidity and mortality (*1*, *2*). A recent analysis of data from 204 countries concluded that multidrug-resistant microorganisms were directly responsible for 1.27 million deaths in 2019 led by resistant strains of *Escherichia coli* and *Staphylococcus aureus* (*3*). Despite this urgent medical need, the discovery and development of new classes of antimicrobials have been relatively stagnant.

Antimicrobial peptides (AMPs) constitute a potential alternative to conventional antibiotics. AMPs are small (10 to 100 amino acids) and often amphipathic host–derived proteins consisting of positively charged residues that intersperse with solvent-exposed hydrophobic amino acids (*4*, *5*). Although, similar to conventional antibiotics, some AMPs may disable intracellular targets, cationic AMPs are thought to kill bacteria primarily by interacting with bacterial anionic membranes (*6*–*8*). The binding of AMPs to bacterial membranes causes membrane disorganization, increased membrane permeability, content leakage, and, ultimately, bacterial death (*6*). Since they target nonprotein structural elements fundamental for bacterial fitness and kill faster compared to conventional antibiotics, AMPs are thought to be less susceptible to bacterial resistance development (*9*, *10*); however, the empiric evidence to support this remains limited.

AMPs constitute an important component of innate immunity and include the cathelicidin, histatin, lectin, and defensin families in humans (*11*, *12*). They promote defense against pathogenic bacteria and may also help shape the microbiome (*13*–*15*). In addition to these professional AMPs, many members of the chemokine superfamily of chemotactic cytokines have been known for decades to have direct antimicrobial activity in vitro (*16*–*18*). Chemokines have many of the biochemical features of AMPs; they are small cationic proteins (7 to 12 kDa), and their family-defining structure contains a C-terminal amphipathic α helix that resembles the structure of many known AMPs (*19*). Of the approximately 50 different mammalian chemokines, over 20 have been shown to kill bacteria, but their antimicrobial mechanisms remain poorly understood (*18*, *20*, *21*).

We recently found that a subset of chemokines binds with high affinity to specific anionic membrane phospholipids, including phosphatidylserine (PS) and cardiolipin (CL) (*22*). PS is typically found in the inner leaflet of mammalian plasma membranes and becomes externalized during apoptosis (*23*, *24*). CL, while present in the inner mitochondrial membrane, is absent in eukaryotic plasma membranes, but it is a common component of the plasma membrane of Gram-negative and Gram-positive prokaryotes (*25*, *26*). We have previously demonstrated that chemokine-PS interactions may play important roles in the regulation of phagocyte recruitment for apoptotic cell clearance (*22*). Here, we investigated the role of chemokine-CL interactions in bacterial killing.

## RESULTS

### Only chemokines that bind anionic phospholipids are antimicrobial

**Fig. 1.**
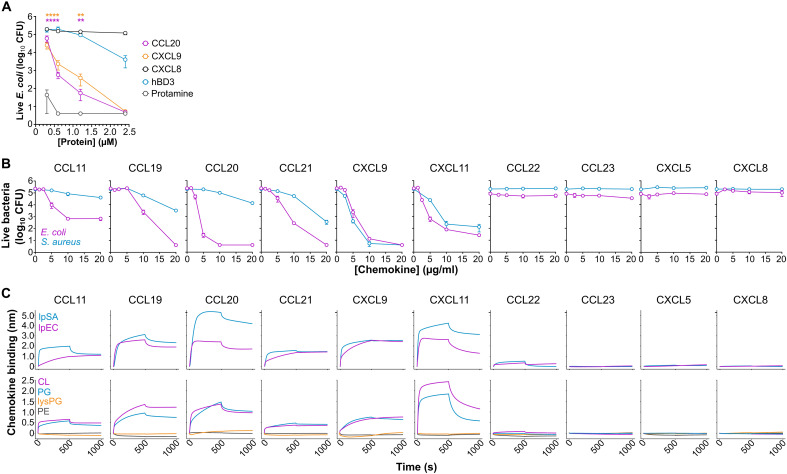
Antimicrobial chemokines bind CL and PG phospholipids. (**A**) Chemokines are more potent antimicrobials than hBD3. Antimicrobial assay showing the number of surviving CFU after incubation (2 hours at 37°C) of 10^+5^ CFU of *E. coli* (strain W3110) with increasing doses of the proteins indicated in the legend in antimicrobial assay buffer (AAB). Data are represented as the means ± SEM CFU of three to five independent experiments analyzed in triplicate. Color-coded asterisks indicate statistically significant differences between each chemokine and hBD3 analyzed by two-way analysis of variance (ANOVA) with Bonferroni multiple comparison test (**P < 0.01; ****P < 0.0001). (**B**) Screening of antimicrobial activity against *E. coli* and *S. aureus* (as coded on the inset of the left-most panel) of increasing doses of the human chemokines indicated above each graph. Experiments were performed and analyzed as in (A). Data are represented as the means ± SD CFU of technical triplicates from one experiment representative of three independent experiments. (**C**) Direct chemokine binding to anionic phospholipid-containing liposomes analyzed by biolayer interferometry (BLI). Top row: BLI sensorgrams showing chemokine binding to liposomes replicating the phospholipid composition of *E. coli* (lpEC; magenta) or *S. aureus* (lpSA; blue). Bottom row: Chemokine binding to phosphatidylcholine (PC) liposomes containing 30% of the phospholipids indicated on the inset of the left graph. Data in (B) and (C) are from one experiment representative of three independent experiments.

Chemokines share many of the biochemical features of cationic AMPs and can have as potent bactericidal activity as classic AMPs (*27*, *28*). Consistent with this, we found that, although less effective than the fish AMP protamine, human CXCL9 and CCL20 killed *E. coli* more potently than human β-defensin 3 (hBD3) (Fig. 1A). This result supports that microbial killing is a bona fide activity of some chemokines. However, the molecular properties that determine which chemokines are antimicrobial remain unknown.

To interrogate whether the mechanism involved chemokine binding to anionic phospholipids, we first tested in a survey of 10 chemokines whether the two activities were correlated, using *E. coli* and *S. aureus* as target organisms. As shown in Fig. 1B, six chemokines—CCL11, CCL19, CCL20, CCL21, CXCL9, and CXCL11—showed clear antimicrobial activity, whereas four chemokines—CCL22, CCL23, CXCL5, and CXCL8—were unable to kill either organism within the tested concentration range (1.25 to 20 μg/ml). Notably, while CXCL9 and CXCL11 displayed similar efficacy against both organisms, CCL11, CCL19, CCL20, and CCL21 killed >1 − log colony-forming units (CFU) of *E. coli* at 5 to 10 μg/ml but required 20 μg/ml to kill >1 − log CFU of *S. aureus* (Fig. 1B).

Next, we used biolayer interferometry (BLI) to analyze binding of the same 10 chemokines to the four main different phospholipids found in bacterial plasma membranes: the anionic phospholipids CL and phosphatidylglycerol (PG), the cationic lysyl-PG (lysPG), and the zwitterionic phosphatidylethanolamine (PE). We tested chemokine binding to these lipids incorporated individually in liposomes of phosphatidylcholine (PC), a zwitterionic phospholipid that does not bind chemokines (*22*), or combined in liposomes replicating the phospholipid composition of the plasma membrane of *E. coli* (lpEC) or *S. aureus* (lpSA), which consists of PE/PG/CL and PG/lysPG/CL, respectively, at an approximate 75/20/5 ratio in both cases (*29*, *30*). As shown in the top row of Fig. 1C, the antimicrobial chemokines CCL11, CCL19, CCL20, CCL21, CXCL9, and CXCL11 bound to both lpEC and lpSA liposomes. Furthermore, these six chemokines bound CL and PG but not PE or lysPG (Fig. 1C, bottom row). In contrast, the nonantimicrobial chemokines CCL22, CCL23, CXCL5, and CXCL8 did not bind any liposome tested (Fig. 1C). These results confirmed CL as a chemokine binding partner, identified PG as a novel lipid binding ligand for chemokines, and demonstrated a strong correlation between chemokine anionic phospholipid binding and antimicrobial activity, in which only chemokines that bound PG and CL were antimicrobial.

### Antimicrobial chemokines bind bacteria through common anionic phospholipid-rich membrane domains

**Fig. 2.**
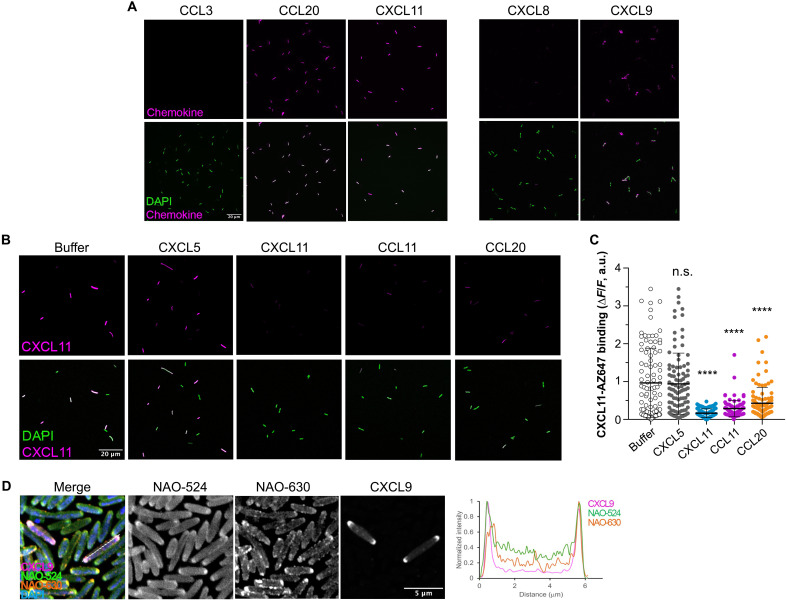
Antimicrobial chemokines share common bacterial binding sites and localize to the cell poles of *E. coli*. (**A**) Antimicrobial chemokines bind directly to bacteria. Representative images of the binding of the AZ647-labeled chemokines (0.3 μM) indicated above each image to *E. coli* (strain W3110). Panels CXCL8 and CXCL9 are separated from the other panels to indicate that these images were acquired with a different microscope (see Materials and Methods). (**B** and **C**) Binding of antimicrobial chemokines to bacteria can be competed with other antimicrobial chemokines. In (B), representative images show the binding of CXCL11-AZ647 (0.3 μM) to *E. coli* (strain W3110) preincubated with buffer (AAB) or the unlabeled chemokines indicated above each image column. In (C), quantification of the fluorescence intensity of CXCL11-AZ647 staining is shown for each treatment group. Each dot corresponds to one bacterium (*n* ≈ 100). Data are from one experiment representative of two independent experiments. Statistical differences between each chemokine group and the “buffer” treatment group were analyzed by ANOVA with Tukey’s test for multiple comparisons (n.s., not significant; ****P < 0.0001). In (A) and (B), the top and bottom image rows show the staining for chemokine alone (magenta) or merged with 4′,6-diamidino-2-phenylindole (DAPI) (green), respectively. A white scale bar (20 μm) is inserted in the bottom left image. a.u., arbitrary units. (**D**) Antimicrobial chemokines bind to the PG/CL-rich membrane domains at the bacterial cell poles. Representative Airyscan confocal images show CXCL9-AZ647 binding to *E. coli* (strain W3110) costained with NAO. Graph on the right shows the normalized fluorescence intensity profile along a bacterium, as indicated by the dashed line in the “merge” panel, of NAO-524 (green), NAO-630 (orange), and CXCL9-AZ647 (magenta).

We next tested the binding of AZ647-labeled chemokines to bacteria. As shown in Fig. 2A, the antimicrobial and PG/CL-binding chemokines CXCL9, CXCL11, and CCL20, but not the nonantimicrobial CXCL8 and CCL3, were able to bind to *E. coli* (*18*, *31*). Consistent with our hypothesis and the results in Fig. 1, we found by BLI that CCL3 does not bind PG or CL (fig. S1). Thus, we concluded that only PG/CL-binding chemokines are able to bind bacteria. Moreover, we found that the binding of one antimicrobial chemokine to bacteria could be competed by a second antimicrobial chemokine but not by a nonantimicrobial chemokine. In particular, as shown in the representative images of Fig. 2B and the quantification of the bacteria-bound CXCL11-AZ647 fluorescence intensity in Fig. 2C, while the binding of CXCL11-AZ647 to *E. coli* was not affected by CXCL5 (nonantimicrobial), it was nearly abolished when the bacteria were preincubated with the unlabeled antimicrobial chemokines CXCL11, CCL11, or CCL20. These results are consistent with a potential role of membrane CL and PG as common binding sites for antimicrobial chemokines in *E. coli*.

CL and PG are known to concentrate at the bacterial cell poles in the plasma membrane of *E. coli* (*32*). This polar localization of CL and PG has been classically investigated by nonyl acridine orange (NAO) staining. NAO is a green fluorophore (NAO-524, λ_em_: 524 nm) that inserts into lipid bilayers, but when it binds to CL, PG, or other anionic phospholipids, its fluorescence emission maximum wavelength (λ_em_) shifts to red (NAO-630, λ_em_: 630 nm) (*32*). Using Airyscan confocal laser scanning microscopy and CXCL9-AZ647 as an example of a fluorescent antimicrobial chemokine, we investigated the localization of bacteria-bound chemokine in *E. coli* costained with NAO. As shown in Fig. 2D, bacteria-bound CXCL9 concentrated and colocalized with NAO-630 at the bacterial poles. Together, these data indicate that antimicrobial chemokines bind to common binding sites localized at PG/CL-rich domains of the bacterial plasma membrane.

### Liposomal anionic phospholipids protect bacteria against antimicrobial chemokines

**Fig. 3.**
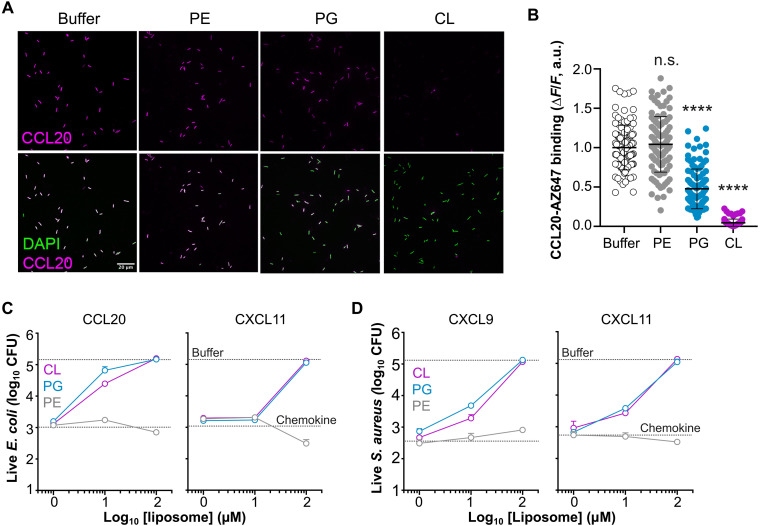
Liposomes containing CL or PG neutralize microbial binding and killing by chemokines. (**A** and **B**) Liposomal PG and CL block chemokine binding to bacteria. Representative images (A) and quantification (B) show the binding of CCL20-AZ647 to *E. coli* (strain W3110) in the presence of PC liposomes (100 μM) containing 30% PE, PG, or CL or buffer alone (AAB) as indicated above each column. Top and bottom image rows show the staining for the chemokine alone or merged with DAPI, respectively. A white scale bar (20 μm) is inserted in the bottom left image. In (B), each dot corresponds to one bacterium (*n* ≈ 100). All liposome-treated groups were compared to buffer by ANOVA with Tukey’s test for multiple comparisons (****P < 0.0001). (**C** and **D**) PG- and CL-containing liposomes protect bacteria from antimicrobial chemokines. Antimicrobial assays show the number of surviving CFU after incubation (2 hours at 37°C) of *E. coli* (C) or *S. aureus* (D) with the chemokines (5 μg/ml) indicated above each graph in the presence of increasing doses of liposomes containing 30% PE, PG, or CL (inset) in AAB. Data are represented as means ± SD CFU from technical triplicates of one experiment representative of three independent experiments. The top and bottom horizontal dotted lines indicate the number of CFU counted when bacteria were incubated with buffer alone or with chemokine in the absence of liposome, respectively.

To investigate the specificity of a possible CL/PG-dependent antimicrobial mechanism by chemokines, we next studied the effect of liposomes of different phospholipid compositions on the ability of chemokines to bind and kill bacteria. For this, we first tested the binding of CCL20-AZ647 to bacteria in the presence of PC liposomes containing PE, PG, or CL. As shown in the representative images in Fig. 3A and the quantification of the fluorescence intensity of CCL20-AZ647 in Fig. 3B, PG- and CL-containing liposomes significantly reduced or blocked, respectively, CCL20-AZ647 binding to *E. coli*, whereas PE-containing liposomes had no effect. Similar results were obtained with AZ647-labeled CXCL11. Although PE reduced the binding of CXCL11-AZ647 to *E. coli* compared to the buffer treatment control, CL and PG completely blocked the binding of CXCL11 to the bacterial surface (fig. S2). We found that liposomes containing CL or PG, but not PE liposomes, protected *E. coli* from the antimicrobial activity of CXCL11 and CCL20 in a dose-dependent manner (Fig. 3C). In particular, 100 μM CL or PG liposomes, corresponding in these experiments to a 1:160 chemokine:lipid molar ratio, sufficed to completely inhibit the killing activity of these two antimicrobial chemokines (Fig. 3C). Furthermore, CL and PG liposomes also neutralized the killing of *S. aureus* by the antimicrobial chemokines CXCL9 and CXCL11 (Fig. 3D). These results confirmed that antimicrobial chemokines bind CL and PG and support that binding to these anionic phospholipids is required for the killing of Gram-negative and Gram-positive microorganisms by chemokines.

### CL-deficient bacteria are more resistant to antimicrobial chemokines

**Fig. 4.**
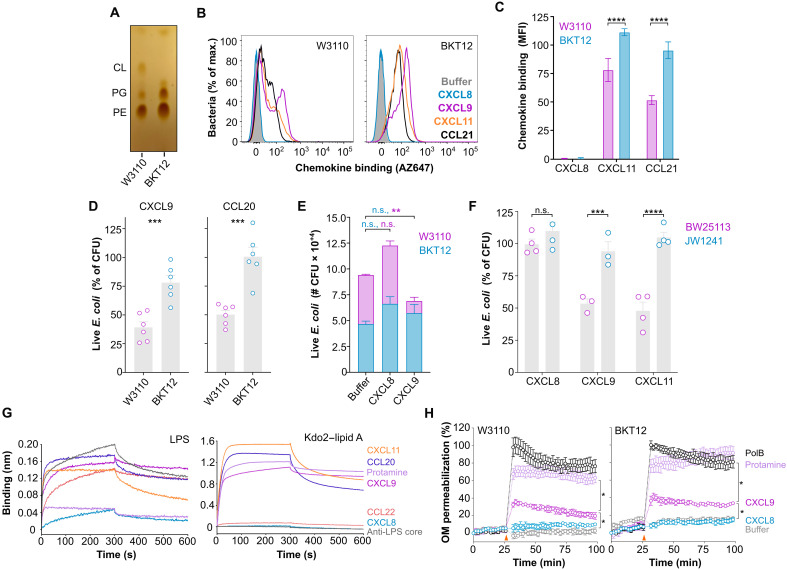
CL-deficient bacteria are resistant to killing but susceptible to OM permeabilization by antimicrobial chemokines. (**A**) TLC showing the phospholipid composition of *E. coli* strains W3110 and BKT12. (**B** and **C**) Binding of fluorescent chemokines (0.3 μM; AAB-85) to W3110 and BKT12. (B) Representative FACS histograms. (C) Quantification of means ± SD median fluorescence intensity (MFI) of biological triplicates from one experiment representative of three experiments. (**D** to **F**) Antimicrobial assays showing the survival of parental (W3110 and BW25113) or CL-deficient (BKT12 and JW1241) *E. coli* strains after incubation (37°C for 2 hours) with chemokines (1.2 μM; AAB-85) or buffer. In (D) and (F), strains (10^+5^ CFU) were treated separately. Bars represent means ± SEM CFU of three to six biological replicates (dots) analyzed in triplicate from two experiments relative (%) to the CFU number in buffer-treated samples. In (E), W3110 and BKT12, mixed 1:1 (10^+5^ total CFU), were treated and plated with and without kanamycin to calculate BKT12 CFU (kanamycin-resistant) and total CFU (W3110 + BKT12). Bars represent means ± SD CFU of biological triplicates analyzed in triplicate from one experiment representative of three experiments. (**G**) BLI sensorgrams showing the binding of the indicated proteins (1 μM) to LPS or Kdo2–lipid A liposomes. (**H**) OM permeabilization assays. NPN fluorescence in W3110 and BKT12 (2 × 10^+7^ CFU) was measured after addition (orange arrowheads) of the indicated proteins (4 μM; AAB-85) or buffer. Results are represented as means ± SEM of biological triplicates relative (%) to the maximum fluorescence obtained with PolB. Data are from one experiment representative of three experiments. Data were analyzed by two-way ANOVA with Bonferroni test (C, E, F, and H) or unpaired *t* test (D). *P < 0.05; **P < 0.01; ***P < 0.001; ****P < 0.0001.

To further assess whether bacterial membrane phospholipids regulate chemokine antimicrobial activity, we next tested the activity of antimicrobial chemokines on the CL-deficient *E. coli* strain BKT12 compared to the wild-type parental strain W3110 (*33*). Using thin-layer chromatography (TLC), we first confirmed the altered phospholipid composition in the BKT12 strain. As shown in Fig. 4A, BKT12 lacked CL and presented increased levels of PG compared to the parental W3110 strain. This heightened ratio of PG in BKT12 has been previously described and attributed to the deletion in this strain of the three CL synthases—ClsA, ClsB, and ClsC—that consume PG to generate CL in *E. coli* (*33*). However, using fluorescence-activated cell sorting (FACS), we found that AZ647-labeled antimicrobial CXCL9, CXCL11, and CCL21 were not only capable of binding to BKT12 bacteria but also displayed a stronger binding to BKT12 than to W3110 (Fig. 4, B and C). Nonantimicrobial CXCL8, which does not bind CL or PG, failed to bind to either *E. coli* strain (Fig. 4, B and C). Therefore, despite the absence of CL in BKT12, antimicrobial chemokines bind W3110 and BKT12 bacteria, which is consistent with the ability of these chemokines to bind both CL and PG phospholipids.

To this point, we had performed all antimicrobial assays in a low-salt buffer [antimicrobial assay buffer (AAB)] commonly used in the field to assess AMP activity. However, we observed that CL-deficient BKT12 bacteria displayed noticeable levels of spontaneous death in low salt, which is consistent with the role attributed to CL in the bacterial response to osmotic stress (*34*). Thus, to avoid any interfering microbial killing by osmotic shock, hereafter, all assays were performed in 85 mM NaCl buffer (AAB-85), which is isosmotic to the bacterial growth medium. Notably, antimicrobial chemokines are known to be sensitive to high salt (*31*); however, we found that this was also applicable to the bona fide AMP hBD3 and that CCL20 and CXCL9 were able to kill >50% CFU in 85 mM NaCl (fig. S3A). CCL20 was still as potent as hBD3 at this higher salt concentration, although 1.2 μM CCL20 was required for a significant antimicrobial effect (fig. S3B). Using these conditions, we found that CXCL9 and CCL20 reduced wild-type W3110 *E. coli* CFU by >50% 2 hours after treatment, whereas they killed only <25% of the CL-deficient BKT12 strain (Fig. 4D). Furthermore, when both strains were incubated simultaneously in a 1:1 W3110:BKT12 CFU mix with chemokines or buffer alone, CXCL9 selectively killed W3110 without affecting the number of BKT12 CFU (Fig. 4E). As expected, the nonantimicrobial chemokine CXCL8 did not reduce the CFU count of either bacterial strain (Fig. 4E). We obtained similar results using a different CL-deficient *E. coli* strain, JW1241, and its parental strain BW25133. As shown in Fig. 4F, the antimicrobial chemokines CXCL9 and CXCL11 killed 50% of BW25133 but had only a marginal effect on the survival of JW1241.

In Gram-negative microorganisms, the plasma membrane is protected by an outer membrane (OM) whose outer leaflet is predominantly composed of lipopolysaccharide (LPS). AMP must either permeate or break the OM to kill bacteria. Although parental and CL-deficient bacterial strains can be expected not to differ in the composition of their OM outer leaflet, to exclude any difference at the OM level that may factor in the heightened resistance of CL-deficient bacteria to chemokines, we next studied LPS binding and OM permeabilization by chemokines. Using BLI, we found that protamine, antimicrobial (CCL20, CXCL11, and CXCL9), and nonantimicrobial (CCL22 and CXCL8) chemokines, as well as an anti-LPS core antibody specific for the oligosaccharide that forms the core domain of LPS, were all able to bind to full-length LPS to some extent (Fig. 4G). In contrast, only the antimicrobial chemokines CCL20, CXCL11, and CXCL9 and protamine were able to interact with liposomal di[3-deoxy-d-manno-octulosonyl]–lipid A (Kdo2–lipid A) (Fig. 4G), a minimal LPS structure containing only the membrane glycolipid moiety. Then, using the fluorescent probe *N*-phenyl-1-phenylnapthylamine (NPN), which fluoresces strongly when it binds to membrane phospholipids but does not cross the hydrophilic barrier formed by LPS on the OM, we analyzed the capacity of chemokines to permeate the OM of W3110 and BKT12 bacteria relative to the well-known OM-active antibiotic polymyxin B (PolB). As shown in Fig. 4H and consistent with their ability to bind Kdo2–lipid A, CXCL9 and protamine, but not the nonantimicrobial chemokine CXCL8, caused significant OM permeabilization. Notably, the ability of protamine to disrupt the OM was comparable to PolB and significantly stronger than that of CXCL9, which may explain its higher antimicrobial efficacy relative to chemokines. W3110 and BKT12 bacteria were equally susceptible to CXCL9-mediated OM permeabilization (~35 to 40%), indicating that the increased chemokine resistance of CL-deficient *E. coli* strains was not due to differences in the chemokine action on the OM. We conclude that antimicrobial chemokines are able to disrupt the OM of *E. coli* and that they are more effective antimicrobials against parental than CL-deficient strains, which supports the role of bacterial membrane CL as a key molecular target for the antimicrobial action of chemokines.

### CL facilitates bactericidal and rapid bacteriostatic action by antimicrobial chemokines

**Fig. 5.**
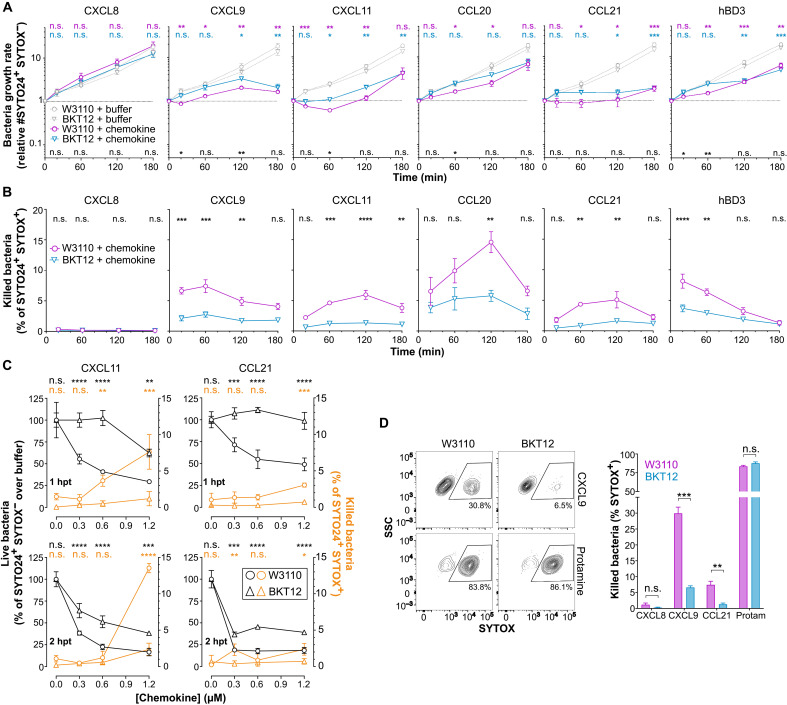
CL promotes rapid bacteriostatic and bactericidal action by antimicrobial chemokines. (**A** and **B**) FACS analysis of the effect of chemokines/hBD3 (1.2 μM; AAB-85) on the growth (A) and killing (B) of W3110 and BKT12 *E. coli* strains over time. The growth ratio was calculated as number of live bacteria (SYTO24^+^ SYTOX^−^)/initial bacterial input. The percentage of killed bacteria (SYTO24^+^ SYTOX^+^) was calculated relative to the number of total bacteria (SYTO24^+^) at each time point. Data are means ± SEM from three to five experiments combined, with biological triplicates in each experiment. Data in (A) were log transformed before statistical analysis. In (A), statistical significances for buffer versus chemokine and W3110 versus BKT12 are color coded above the graphs or above the *x* axes, respectively. (**C**) Live (black lines) and dead (orange lines) W3110 and BKT12 bacteria 1 or 2 hpt with increasing doses of CXCL11 and CCL21. Live bacteria (left *y* axis) posttreatment are represented relative (%) to the number of live bacteria in buffer-treated samples. The percentage of killed bacteria (right *y* axis) was calculated as in (B). Data are means ± SD of biological triplicates from one experiment representative of three experiments. Statistical significances of the bacteriostatic and killing activity are color coded above the graphs. (**D**) Right: Quantification of the percentage of killed (SYTOX^+^) W3110 or BKT12 bacteria after treatment with the indicated proteins (4.8 μM; AAB-85; 20 min). Bars show the means ± SD of biological triplicates from one experiment representative of three experiments. Left: Representative FACS contour plots. Data were analyzed by two-way ANOVA with Tukey (A) or Bonferroni (B and C) test or by multiple *t* test (D). *P < 0.05; **P < 0.01; ***P < 0.001; ****P < 0.0001. SSC, side scatter; Protam, protamine.

To gain further insight into the chemokine antimicrobial mechanism, we next performed a FACS-based time-to-kill assay. For this, W3110 and BKT12 bacteria were treated with a fixed dose (1.2 μM) of chemokines, and live and dead bacteria were quantified at 20, 60, 120, and 180 min after treatment by nucleic acid staining with SYTOX and SYTO24. SYTOX only permeates and stains dead bacteria with compromised plasma membrane integrity, whereas SYTO24 detects both live and dead bacteria. For reference, hBD3 was included in these experiments. Using this assay to quantify the number of live bacteria (SYTO24^+^ SYTOX^−^), we found that W3110 and BKT12 treated with buffer alone or the nonantimicrobial chemokine CXCL8 grew at similar rates during the 3-hour experiment (Fig. 5A). By the end of the experiment, the number of live bacteria in the CXCL8- or buffer-treated samples multiplied by nearly 20-fold relative to the initial bacterial input (Fig. 5A). In contrast, all tested antimicrobial chemokines—CXCL9, CXCL11, CCL20, and CCL21—and hBD3 stopped or significantly slowed bacterial growth (Fig. 5A). This differed from the pronounced drop in the number of live bacteria observed after incubation with protamine (fig. S4). All antimicrobial chemokines stalled the growth of the parental W3110 strain to a larger extent and at earlier times than that of the CL-deficient BKT12 strain (Fig. 5A). For instance, CXCL9 and CCL20 significantly decelerated the replication of W3110 bacteria within 20 or 60 min after treatment, respectively, whereas BKT12 bacteria treated with these chemokines grew at a rate comparable to buffer-treated BKT12 for almost 2 hours (Fig. 5A). This was consistent with the different susceptibility of these two strains to CXCL9 and CCL20 after 2 hours of treatment shown in Fig. 4D. Similarly, both CCL21 and CXCL11 inhibited the replication of W3110 to a greater degree than that of BKT12 (Fig. 5A). The BKT12 strain was not completely resistant to antimicrobial chemokines or hBD3. By 3 hours after treatment, all antimicrobial proteins were capable of controlling the growth of this CL-deficient strain (Fig. 5A), possibly via their interaction with PG on the membrane of BKT12 or some other mechanism. However, these data support that membrane CL facilitates prompt control of bacterial replication by antimicrobial chemokines. Furthermore, perhaps with the exception of CXCL9 and CCL21, chemokine- and hBD3-treated bacteria seemed to overcome the initial growth retardation at later times. This highlights the importance of these time-course experiments versus the static observation obtained at one time point by CFU analysis. On the other hand, although chemokines appeared to be mainly bacteriostatic at this dose (1.2 μM), when we analyzed the presence of dead bacteria (SYTO24^+^ SYTOX^+^) at each time point, we found that W3110 was significantly more susceptible than BKT12 to direct plasma membrane permeabilization by all antimicrobial chemokines and hBD3 (Fig. 5B). As expected, dead bacteria were not found when either bacterial strain was treated with the nonantimicrobial chemokine CXCL8 (Fig. 5B). Together, these data indicate that chemokines can exert bacteriostatic and bactericidal effects and that both mechanisms are promoted by bacterial membrane CL.

One caveat to our interpretation of the time-to-kill data is that in these experiments the bacteriostatic and bactericidal activities may influence each other. For instance, the bactericidal effect may reduce the total number of live bacteria and, as a result, decelerate the growth of the overall population. Therefore, we next attempted to uncouple these two mechanisms to clearly resolve the role of membrane CL in the antimicrobial activity of chemokines. For this, we first performed time-to-kill assays with decreasing amounts of chemokine aiming to detect bacteriostatic effects at low nonbactericidal doses of chemokine. As shown in Fig. 5C and consistent with the data in Fig. 5B, high doses of CXCL11 and CCL21 (>0.6 μM) displayed detectable and significant levels (up to 14% SYTOX^+^ cells) of direct killing activity (orange lines) against W3110 but only marginal bactericidal activity (<2% SYTOX^+^ cells) against the CL-deficient BKT12 strain at 1 and 2 hours posttreatment (hpt). Subthreshold concentrations for bacterial killing (0.3 μM) of CXCL11 and CCL21 reduced the number of live W3110 bacteria (black lines) to 50 and 70%, respectively, relative to the buffer-treated group 1 hpt, whereas they did not impair the growth of the BKT12 strain at this early time point (Fig. 5C, top). Notably, although the bacteriostatic effect against BKT12 of both chemokines at all doses became apparent 2 hpt, the growth of W3110 was still more severely reduced at this later time (Fig. 5C, bottom). Therefore, while not categorically required for antimicrobial chemokines to control the growth of *E. coli* eventually, we concluded that bacterial membrane CL facilitates rapid onset of chemokine-mediated bacteriostatic effects.

On the other hand, to confirm the role of CL in bacterial killing by chemokines without interference of their bacteriostatic effects, we next analyzed the number of dead W3110 and BKT12 bacteria shortly after treatment with a high dose of antimicrobial chemokine. For this, bacteria were incubated with 4.8 μM CXCL9, CCL21, CXCL8, or the bactericidal peptide protamine, and the percentage of SYTOX^+^ cells was analyzed 20 min after treatment by FACS. As shown in Fig. 5D, CXCL9 killed ~30% of W3110 bacteria but only 6% of BKT12, whereas CCL21 killed 8 and 1%, respectively. In contrast, protamine was equally effective against both strains and killed about 85% of W3110 and BKT12 bacteria (Fig. 5D), proving that the BKT12 strain was not inherently more resistant to plasma membrane permeabilization by AMPs. As expected, CXCL8 did not kill either bacterial strain. At this early time, CXCL9 killed considerably more W3110 bacteria than CCL21; however, as shown in Fig. 5B, these two chemokines appear to kill with different kinetics (peak killing at ~20 min or 2 hours after treatment, respectively). Accordingly, when we analyzed the percentage of SYTOX^+^ bacteria 90 min after treatment, the ratio of W3110 bacteria directly killed by CCL21 increased to 14%, whereas it killed only 3% of BKT12 (fig. S5). These results demonstrate that the CL-deficient BKT12 strain is more resistant to plasma membrane permeabilization by antimicrobial chemokines.

### Antimicrobial chemokines lyse membranes containing anionic phospholipids

**Fig. 6.**
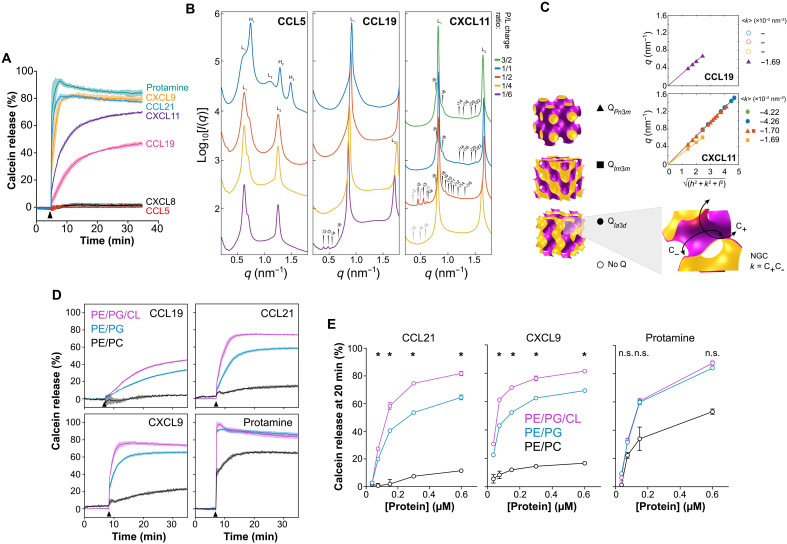
Antimicrobial chemokines lyse phospholipid bilayers in an anionic phospholipid-dependent manner. (**A**) Calcein-leakage assay showing the release of calcein from PE/PG/CL liposomes upon injection (arrowhead) of chemokines/protamine (1.2 μM). Curves represent the percentage of calcein released relative to the maximum release observed with 0.1% Triton X-100. Solid lines represent the mean of biological triplicates. Colored shaded area represents the SD. Data are from one experiment representative of three experiments. (**B**) Radially integrated SAXS spectra of chemokines interacting with 20/80 PG/PE liposomes at increasing (1/6 to 3/2) peptide-to-lipid (P/L) charge ratios. Bragg structure peaks were indexed for the observed phases: cubic (Q) (arrowed indices), lamellar (L), and inverted hexagonal phases (H). (**C**) Linear fits of the peak position for the cubic phases, *Pn*3*m*, *Im*3*m*, and *Ia*3*d* (illustrated next to symbol key), indexed in (B). Estimation of mean NGC, <*k*>, from fits is displayed next to each plot. Colors represent each P/L ratio as noted in (B). (**D**) Calcein-leakage assays showing the percentage of calcein released from three types of liposomes (inset of CCL19 panel) after injection (arrowheads) of the indicated proteins (1.2 μM). Data were analyzed as in (A) and correspond to biological triplicates from one experiment representative of three experiments. (**E**) Quantification of the percentage of calcein released from different liposomes (inset of CXCL9 panel) 20 min after addition of increasing doses of the indicated proteins. Data are means ± SD of biological triplicates from one experiment representative of three experiments. Data were analyzed by two-way ANOVA with Tukey test. Statistical differences (PE/PG/CL versus PE/PG) are indicated above the graphs. *P < 0.05.

To confirm the membrane lytic activity of chemokines in a more direct way, we next performed liposome calcein leakage assays. In these assays, the fluorescent dye calcein is self-quenched when trapped at high concentrations inside liposomes, but it fluoresces when released and diluted into the extraliposomal medium after a membrane active peptide ruptures the liposomal membrane. Consistent with their bactericidal activity, we found that CCL19, CCL21, CXCL9, and CXCL11 as well as protamine were able to lyse liposomes that replicated the phospholipid composition of W3110 bacteria (PE/PG/CL, 75/20/5 mass ratio) (Fig. 6A). In contrast, the nonantimicrobial chemokines CXCL8 and CCL5 had no effect on the permeability of these liposomes (Fig. 6A). We previously demonstrated that CCL5 does not bind CL or PG (*22*) and, consistent with our previous data, unlike CCL19, CCL5 is innocuous to bacteria (fig. S6A).

To assess the ability of antimicrobial chemokines to disrupt membranes in a manner analogous to pore formation processes used by membrane active cationic AMPs (*35*, *36*), we performed high-resolution synchrotron small-angle x-ray scattering (SAXS) experiments to characterize the membrane remodeling of the chemokines on bilayer membranes. In agreement with their antimicrobial activity, we found that CCL19 and CXCL11 remodeled spherical PG-containing liposomes into negative Gaussian curvature (NGC) rich cubic phases (Fig. 6, B and C). This membrane remodeling geometry is necessary for the restructuring of the membrane surface during pore formation, which is characteristic of cationic amphipathic AMPs (*35*, *36*). Furthermore, consistent with its stronger liposomal membrane lytic activity (Fig. 6A), CXCL11 induced stronger NGC curvatures than CCL19, up to 4.26 × 10^−2^ nm^−2^ (Fig. 6C). In contrast, CCL5, a nonantimicrobial chemokine, did not induce NGC surface remodeling (Fig. 6B). Using estimates based on membrane mechanical elasticity for a typical membrane (charge density = −0.05 A·s/m^2^, Debye length = 1 nm, and line tension = 10 pN), we can infer from the SAXS measurements that the NGC induced by CXCL11 and CCL19 corresponds to the formation of a transmembrane pore with a diameter of 2.4 to 2.9 nm and 2.4 to 2.5 nm, respectively (*37*).

To understand the importance of each bacterial phospholipid for the membrane disrupting action of antimicrobial chemokines, we next performed liposome calcein leakage assays with PE/PG/CL liposomes (75/20/5, mass ratio), CL-lacking PE/PG liposomes (75/25, mass ratio), which mimic the phospholipid composition of the plasma membrane of the BKT12 *E. coli* strain, and PE/PC (75/25, mass ratio) liposomes lacking all anionic phospholipids. As shown in Fig. 6D, CCL19, CCL21, and CXCL9 (1.2 μM) released minimum levels of calcein from PE/PC liposomes and permeabilized PE/PG/CL liposomes more effectively than PE/PG liposomes. In contrast, protamine was equally effective at releasing calcein from PE/PG/CL and PE/PG liposomes (Fig. 6D). Similar results were obtained with a much lower dose (0.15 μM) of antimicrobial protein (fig. S6B). We confirmed these observations in dose-response calcein leakage assays using the three different types of liposomes. Increasing doses of CXCL9 and CCL21 failed to permeabilize PE/PC liposomes and released significantly lower levels of calcein from PE/PG than those from PE/PG/CL liposomes, whereas protamine leaked comparable levels of calcein from PE/PG and PE/PG/CL liposomes at all doses (Fig. 6E). Hence, the presence of CL in the liposomes was irrelevant for the membrane lytic activity of protamine, whereas it facilitated membrane disruption by chemokines. These results aligned with our findings that W3110 and BKT12 are equally susceptible to membrane permeabilization by protamine but the CL-deficient strain is more resistant to plasma membrane disruption by antimicrobial chemokines (Fig. 5D). We conclude that the membrane lytic activity of chemokines requires the presence of anionic phospholipids, particularly CL.

### Bacteria fail to develop resistance against antimicrobial chemokines

It is thought that AMPs are less susceptible to antimicrobial resistance due to their rapid action and membrane-attacking mechanism (*10*). However, this has been experimentally demonstrated for very few peptides and not for chemokines. Since our data support that antimicrobial chemokines act quickly and target bacterial membranes, we next investigated whether bacteria develop resistance to antimicrobial chemokines.

**Fig. 7.**
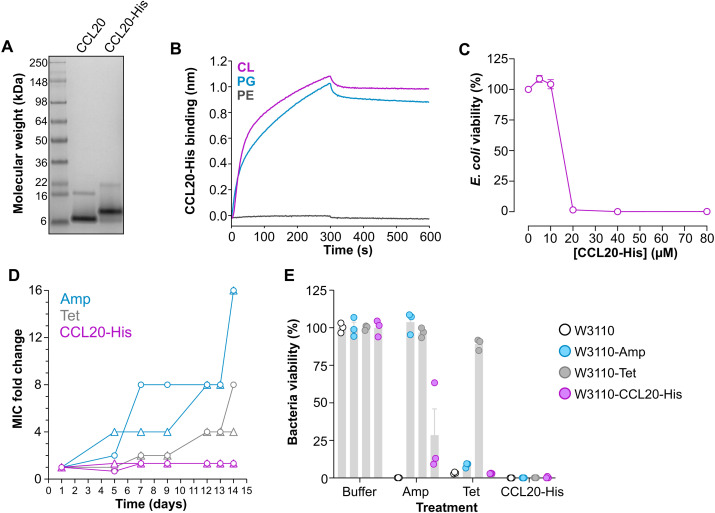
CCL20 kills parental and antibiotic-resistant *E. coli* without triggering chemokine resistance. (**A**) Coomassie Blue–stained gel for commercially available CCL20 (Peprotech) and in-house produced CCL20-His. (**B**) BLI assays showing the binding of CCL20-His to liposomes containing 30% of the indicated phospholipids (inset). (**C**) Determination of the MIC of CCL20-His. *E. coli* (W3110 strain) bacteria were incubated (18 hours at 37°C) with increasing doses of CCL20-His in MHB, and the percentage of viable bacteria relative to bacteria treated with buffer alone was calculated using the BacTiter-Glo Kit. Graph shows means ± SEM of bacterial viability (%) from four experiments combined analyzed in duplicate. (**D**) Bacteria develop resistance against conventional antibiotics but not against CCL20-His. W3110 bacterial cultures were maintained in MHB for 2 weeks in the presence of a sublethal dose (0.5 × MIC) of tetracycline (Tet), ampicillin (Amp), or CCL20-His, as indicated on the inset. On selected days, MIC for each antimicrobial agent with the corresponding culture was recalculated, and bacteria were subcultured adjusting the agent dose to the new MIC. Graphs show the MIC fold change relative to the initial MIC of two independent bacterial cultures, represented by circle and triangle symbols, for each agent. (**E**) CCL20-His kills Tet- and Amp-resistant bacterial strains. Parental W3110 bacteria (5 × 10^+5^ CFU/ml) or the conditioned bacterial strains generated in (D), W3110-Amp, W3110-Tet, or W3110-CCL20-His, were incubated (18 hours at 37°C) in MHB with Amp, Tet, or CCL20-His at a concentration equivalent to their original MIC. Bacterial viability in each sample was calculated as in (C). Bars represent means ± SEM of bacterial viability (%) of biological triplicates from one experiment representative of three experiments.

For this, we purified a C-terminally His-tagged form of CCL20 (CCL20-His), which was refolded from insoluble and unfolded protein obtained by expression in bacteria (Fig. 7A). We first characterized the lipid-binding properties of CCL20-His by BLI and confirmed that this chemokine killed bacteria. As shown in Fig. 7B, similar to untagged CCL20 (Fig. 1C), CCL20-His bound PG and CL but not PE. Then, we calculated the minimum inhibitory concentration (MIC) of CCL20-His against the wild-type *E. coli* strain W3110 in Mueller-Hinton broth (MHB) by the microdilution method. We found that the MIC of CCL20-His was 20 μM (Fig. 7C), which is in line with the MIC of other AMPs and chemokine-derived peptides (*38*–*40*). These results confirmed that CCL20-His was an active antimicrobial chemokine.

To study whether *E. coli* develops resistance to CCL20-His, we maintained W3110 bacteria in MHB in the presence of a CCL20-His concentration equivalent to 0.5 × MIC for 2 weeks. For comparison, the conventional antibiotics ampicillin (Amp) and tetracycline (Tet) were also included in this experiment, whose initial MICs, calculated as in Fig. 7C, were 5 and 0.5 μg/ml (or 14.3 and 1.1 μM), respectively. On selected days, the MICs of all three compounds were recalculated to assess the evolution of the MIC and to readjust the treatment if necessary to the new MIC. Two separate cultures were initiated and maintained for each antimicrobial compound and analyzed independently. On day 7, the Amp MIC in both cultures treated with Amp increased by >4-fold and surged to 228 μM (16-fold change) by the end of the experiment (Fig. 7D), indicating that bacteria had become resistant to Amp. Although at a slower rate, the Tet MIC also increased and reached 8.8 and 4.4 μM, an eight- and fourfold increase, for the two separate Tet-treated cultures, respectively, on day 14. In notable contrast, the CCL20-His MIC remained steady at 20 μM throughout the duration of the experiment (Fig. 7D), indicating that *E. coli* failed to develop resistance against this chemokine. The resulting bacterial cultures after the 14-day conditioning with Amp, Tet, or CCL20-His, were collected, named W3110-Amp, W3110-Tet, and W3110-CCL20-His, respectively, and then challenged with a 1 × MIC dose of these three compounds. As shown in Fig. 7E, consistent with the acquired resistance, Amp and Tet killed the parental W3110 and the W3110-CCL20-His strains but were inactive on W3110-Amp or W3110-Tet, respectively. Notably, the W3110-Tet strain was also resistant to Amp, which is consistent with reports of cross-resistance observed in Tet-resistant bacterial strains (*41*). CCL20-His killed the parental and all three W3110 conditioned strains (Fig. 7E), which indicates that this chemokine can circumvent Amp and Tet resistance mechanisms to kill bacteria. Furthermore, we found that other antimicrobial chemokines were also able to kill antibiotic-resistant strains effectively. For instance, we found that W3110 and W3110-Amp strains were equally susceptible to CXCL9 (fig. S7). These results prove that antimicrobial chemokines kill bacteria without triggering microbial resistance and that they inactivate antibiotic resistant microorganisms.

## DISCUSSION

In this study, we demonstrate that chemokine antibacterial activity requires chemokine binding to anionic membrane phospholipids. We show that PG/CL-binding activity is required for microbial killing by chemokines and that all chemokines tested that failed to bind to these membrane anionic phospholipids lacked antimicrobial activity, whereas all those tested that had PG/CL-binding activity were antimicrobial. Furthermore, we prove that bacterial membrane CL mediates rapid onset of chemokine bacteriostatic and bactericidal effects. We show that CL-deficient bacteria are more resistant to growth arrest and membrane permeabilization by chemokines and that antimicrobial chemokines selectively target CL-containing bacteria when these are mixed with CL-deficient bacteria. We found that bacteria failed to develop resistance against antimicrobial chemokines in our experiments. Since CL is an essential anionic phospholipid in the membranes of most Gram-negative and Gram-positive bacteria, our study provides proof of principle for the development of chemokines as broad-spectrum antimicrobials resistant to antimicrobial resistant mechanisms.

It has been known that bacterial plasma membrane components and anionic phospholipids, in particular, are interaction partners for many classic AMPs (*6*, *8*). However, despite existing evidence of direct bacterial membrane disruption by chemokines (*42*, *43*), this has sometimes been diminished as a secondary or nonspecific killing mechanism (*21*). Furthermore, the contribution of bacterial membrane anionic phospholipids to this chemokine activity has been overlooked. Recently, we found that many but not all human chemokines are able to bind PS and CL with high affinity (*22*). The biological importance of PS to apoptotic cells and apoptotic bodies and the selective localization of CL to bacterial plasma membranes motivated hypotheses for the biological significance of chemokine binding to these phospholipids. In this regard, we have previously reported that chemokine binding to PS may be a “find-me” signal for apoptotic cell clearance by macrophages (*22*, *44*). In the present work, we demonstrate that antimicrobial chemokines bind three types of negatively charged bacterial lipids: CL, PG, and lipid A. We show that, while their interaction with lipid A allows chemokines to disrupt the OM, ultimately, binding of chemokines to CL is intrinsic to their mechanism of antimicrobial activity since CL-deficient bacteria strains were more resistant to chemokines, particularly at early times. We found that not all chemokines bind anionic lipids but all that bind PS also bind CL, PG, and lipid A. However, our work also supports some degree of molecular specificity for chemokine-lipid binding that indicates that these interactions are not solely driven by charge. For example, we previously reported that chemokines selectively bind CL and PS over other more highly anionic phospholipids and that some highly cationic chemokines fail to interact with either anionic phospholipid (*22*). Furthermore, with the exception of CCL3, all the chemokines and AMP tested in this study have a basic isoelectric point and high content of basic residues, but not all of them bind lipids or kill bacteria (table S1). This agrees with previously published studies that excluded the isoelectric point of chemokines as a reliable predictor of antimicrobial activity (*18*, *19*). Similarly, classic AMPs are generally cationic and amphiphilic but are not limited to these physicochemical properties (*4*, *36*). Here, we demonstrate that the property that may define which chemokines are antimicrobial and which are not is their ability to bind CL- and PG-containing membranes. The precise molecular and chemical properties that allow some chemokines to interact with these anionic phospholipids and, in turn, kill bacteria, will require further investigation.

Our findings do not exclude the possibility that, as reported for other AMPs (*7*), chemokines may use more than one antimicrobial mechanism, including targeting bacterial proteins or DNA to kill bacteria. In this regard, the transmembrane adenosine 5′-triphosphate–binding cassette transporter permease FtsX, the pyruvate dehydrogenase complex (PDHc), and the adenosine 5′-triphosphate–binding cassette transport system Opp have been reported to facilitate the killing of *Bacillus anthracis*, *E. coli*, and *Streptococcus pneumoniae*, respectively, by CXCL9, CXCL10, and CXCL11 (*45*–*47*). However, direct interaction of these chemokines with FtsX, PDHc, or Opp has not been demonstrated. A 27–amino acid region in an external loop of FtsX was initially proposed as a putative binding site in *B. anthracis* for CXCL9, CXCL10, and CXCL11 due to its similarity with the N-terminal chemokine-binding domain of CXCR3, the human cellular receptor for these three chemokines (*45*). Nevertheless, this short region of FtsX shares only 22% amino acid identity with the equivalent region on the N terminus of CXCR3, and it has been shown that FtsX is dispensable for the killing of *Bacillus subtilis* and *S. pneumoniae* by CXCL10 (*47*). Moreover, it has been reported that functional FtsX and PDHc rather than their presence are required for CXCL10-mediated killing of *B. anthracis* and *E. coli*, respectively (*46*, *48*, *49*). FtsX, PDHc, and Opp regulate cell division and peptidoglycan synthesis, the conversion of pyruvate to acetyl coenzyme A, and the uptake of oligopeptides involved in nutrition and cell-to-cell communication, respectively, all essential processes for the overall energetic and metabolic state of bacteria (*50*–*53*). Therefore, it may be reasonable to propose that, rather than acting as direct chemokine targets, FtsX, PDHc, and Opp may indirectly control bacterial permeability and membrane homeostasis, which ultimately may impair membrane binding and membrane disruption by chemokines or other AMPs. Consistent with this, Opp- and FtsX-deficient bacteria have been shown to be also partially resistant to other membrane active peptides such as nisin and LL-37 (*47*). Furthermore, it is important to remember that >20 different chemokines are known to be antimicrobial and that chemokines kill a wide range of bacterial species including *E. coli*, *Klebsiella pneumoniae*, *Pseudomonas aeruginosa*, *S. aureus*, *Streptococcus pyogenes*, *S. pneumoniae*, and others (*18*, *21*). Therefore, a binding site common to all antimicrobial chemokines and conserved across Gram-negative and Gram-positive bacteria, such as CL or PG, may be more likely than the existence of a species-specific bacterial protein ligand for each chemokine. Here, we provide several lines of evidence supporting that the binding to PG and, particularly, CL constitute an intrinsic component of the antimicrobial mechanism of chemokines: (i) All antimicrobial chemokines tested in our study (6 of 6) bind PG and CL, whereas all nonantimicrobial chemokines included here (6 of 6) failed to bind these anionic phospholipids; (ii) the binding of CXCL11 to *E. coli* can be blocked by other antimicrobial chemokines, regardless of their different cellular receptors and other biochemical differences, supporting that different antimicrobial chemokines interact with the same bacterial binding sites; (iii) bacteria-bound antimicrobial chemokines localize to the poles of the bacterial cell, a region of the *E. coli* plasma membrane where PG and CL phospholipids are known to concentrate (*32*); (iv) CL-deficient *E. coli* are more resistant to growth arrest and membrane permeabilization by antimicrobial chemokines; and (v) the presence of PG or CL is required for bilayer membrane disruption by antimicrobial chemokines, with CL playing a bigger role than PG in membrane lysis by chemokines but not by other AMPs such as protamine. Although multifunctional molecules such as chemokines can be expected to be able to exert different killing mechanisms in different contexts and it has been reported that some chemokines can act as bifunctional antimicrobial agents (*49*), we propose that CL/PG-binding activity of antimicrobial chemokines and derived variants should be tested before establishing membrane-independent bacterial killing mechanisms.

We also show here that antimicrobial chemokines can exert bactericidal and bacteriostatic effects and that both antimicrobial effects are promoted by bacterial membrane CL. We found that the absence of CL significantly impaired the ability of antimicrobial chemokines to disrupt phospholipid liposomes and the bacterial plasma membrane and it delayed chemokine-induced bacterial growth arrest. We show that antimicrobial chemokines, but not nonantimicrobial chemokines, are able to lyse liposomes containing a phospholipid composition similar to that of the plasma membrane of *E. coli* (PE/PG/CL) but failed to disrupt liposomes lacking PG and CL. Furthermore, SAXS measurements demonstrated the ability of antimicrobial chemokines to remodel PG-containing liposomal membranes into NGC-rich surfaces, a geometry necessary for membrane permeabilization and pore formation (*35*, *36*). Understanding the molecular mechanisms by which phospholipid-binding chemokines halt bacterial growth will require further investigation. Anionic phospholipids, and particularly CL, are known to play major roles in bacterial replication by acting as anionic scaffolds in the bacterial plasma membrane for proteins, protein complexes, and DNA during cell division (*25*, *54*, *55*). It is possible that CL-binding chemokines may interfere with the recruitment of these essential elements of the bacterial replication machinery to membrane CL microdomains, ultimately stalling bacterial replication. Another possibility is that similar to the bacteriostatic mechanism of other AMPs such as buforin II or indolicidin (*56*, *57*), antimicrobial chemokines may permeate the bacterial plasma membrane to interact with and destabilize the bacterial genome. Consistent with this hypothesis, some chemokines are known to bind DNA directly (*42*, *58*). In addition, more experimentation will be needed to understand why CL-deficient PG-containing bacteria grow normally early after chemokine challenge but succumb later. In this regard, it would be interesting to investigate the effect of antimicrobial chemokines on PG-deficient bacteria. However, *E. coli* strains lacking PG are not viable unless the major OM lipoprotein Lpp is also deleted (*29*, *59*), which may add uncontrollable effects on fitness and on the mechanical properties of the bacterial membranes (*60*), potentially affecting the sensitivity of these strains to membrane active AMPs and making comparisons with PG-containing bacteria problematic.

The results presented here have implications for understanding the role of endogenous AMPs in innate immunity and for the potential development of antimicrobial chemokines as a new class of antibiotic. To date, of the more than 3000 found AMPs, only nine (daptomycin, colistin, vancomycin, telavancin, teicoplanin, bacitracin, dalbavancin, oritavancin, and gramicidin) have been approved by the US Food and Drug Administration for clinical use (*61*, *62*). In part, the difficulty in testing an in vivo physiologic role for any AMP relates to the large number of known endogenous AMPs and the potential for redundant action (*63*). Nevertheless, some existing data support that chemokines may exert antimicrobial effects in vivo. For instance, some chemokines are expressed at high levels in certain barrier tissues without causing inflammation, such as CCL28 or CXCL17 in saliva and CCL20 in skin and Peyer’s patches (*64*–*67*). CXCL9-depleted mice have been shown to be more susceptible to *Citrobacter rodentium* and *B. anthracis* in a manner that is independent of its receptor CXCR3 (*43*, *68*). Although our data show that chemokines need a fairly high concentration to exert bactericidal effects (>1.2 μM) or reach their MIC (20 μM for CCL20-His), which may be difficult to achieve in vivo, it is possible that endogenous antimicrobial chemokines may act in concert with other AMPs, such as cathelicidins or defensins, or through their bacteriostatic activity, which, we show here, can be effective at submicromolar concentrations (<0.3 μM), especially if the target bacteria contain CL. On the other hand, while this concentration-related issue might be easy to overcome in a therapeutic application, a major challenge for the use of all AMPs in the clinic is their sensitivity to salt concentrations (*69*). Although not all tissues and secretions have the same salt content (*31*) and a comprehensive analysis of the salt sensitivity of the >20 different antimicrobial chemokines is lacking, it would be desirable to engineer salt-insensitive chemokine variants. In this regard, certain residue modifications have been known to improve the salt resistance of AMPs (*70*, *71*). Our data support that if similar alterations were made to chemokines, then the variants should preserve their PG- and CL-binding activity. Another limitation for the clinical application of AMPs is their immunogenicity and low stability in vivo (*72*). In contrast, since chemokines are host proteins, they should be nonimmunogenic. However, many have a short half-life in blood, potentially driven by serum protease action and by scavenging by the erythrocyte atypical chemokine receptor 1, cognate leukocyte chemokine receptors and glycosaminoglycans (*73*, *74*). Therefore, precise mapping of the CL/PG-binding sites in chemokines may guide the creation of antimicrobial chemokine variants with improved bioavailability that could serve as an alternative therapeutic approach to treating bacterial infections while preventing the generation of antibiotic-resistant bacteria.

In summary, we have provided evidence that supports specific anionic phospholipid binding as an important component of the microbial killing mechanism that defines which chemokines are antimicrobial and which are not. We have recently characterized the importance of chemokine interactions with another anionic phospholipid, PS, for phagocyte recruitment in the context of apoptosis (*22*). Here, we show that binding to PG and particularly CL is important for microbial killing by chemokines. Together, the present study and our previous study demonstrate the potential physiological relevance of chemokine-phospholipid interactions.

## MATERIALS AND METHODS

### Reagents and bacteria strains

Unlabeled recombinant chemokines and hBD3 were purchased from Peprotech (Rocky Hill, NJ) and R&D Systems (Minneapolis, MN), respectively. AZ647-labeled chemokines were acquired from Protein Foundry (Milwaukee, WI).

*S. aureus* strain Wichita (ATCC 29213) was purchased from American Type Culture Collection (ATCC; Manassas, VA). *E. coli* parental strains, W3110 and BW25113, and their CL-deficient mutants, BKT12 and JW1241, respectively, were acquired from the Coli Genetic Stock Center at Yale University (New Haven, CT). The BKT12 strain was generated by Tan *et al.* (*33*).

### Antimicrobial assays

*S. aureus* and *E. coli* were grown overnight in tryptic soy broth (TSB) at 37°C. The CL-deficient strains BKT12 and JW1241 were grown in the presence of kanamycin (50 μg/ml). TSB and kanamycin were purchased from KD Medical (Columbia, MD). Stationary cultures were diluted 1:100 in TSB and grown to mid-early log phase [optical density at 600 nm (OD_600_) = 0.4 to 0.6]. Bacteria were collected by centrifugation (2500*g* for 5 min) and washed once with AAB [10 mM tris-HCl (pH 7.4) and 1% TSB]. Where indicated, AAB was supplemented with 85 mM NaCl (AAB-85). Chemokines and other AMPs were incubated with 1 × 10^+5^ CFU of bacteria in 100 μl of AAB for 2 hours at 37°C. Bacterial viability was then tested by CFU determination. For this, serial 10-fold dilutions were plated on agar plates in triplicate. Plates were incubated at 37°C overnight, and visible CFU were counted manually. Where indicated, BacTiter-Glo Microbial Cell Viability Assay (Promega, Madison, WI) was used instead to determine bacterial viability.

### Liposomes

Phospholipid liposomes were prepared by the extrusion method. All lipids used in this study—1,2-dioleoyl-*sn*-glycero-3-phosphocholine (DOPC or PC), 1,2-dioleoyl-*sn*-glycero-3-phospho-(1′-rac-glycerol) (DOPG or PG), 1,2-dioleoyl-*sn*-glycero-3-phosphoethanolamine (DOPE or PE), 1′,3′-bis[1,2-dioleoyl-*sn*-glycero-3-phospho]-glycerol (CL), 1,2-dioleoyl-*sn*-glycero-3-[phospho-rac-(3-lysyl(1-glycerol))] (lysPG), 1,2-distearoyl-*sn*-glycero-3-phosphoethanolamine-*N*-[biotinyl(polyethylene glycol)-2000] (DSPE-PEGbiot), and Kdo2–lipid A—were purchased from Avanti Polar Lipids (Alabaster, AL). When used for BLI experiments, “PE,” “PG,” “lysPG,” and “CL” liposomes contained 30% of the corresponding phospholipid, 65% PC, and 5% DSPE-PEGbiot (weight %); “Kdo2–lipid A” liposomes contained 50% Kdo2–lipid A, 45% PC, and 5% DSPE-PEGbiot (weight %); and “lpSA” and “lpEC” liposomes consisted of PG/lysPG/CL/DSPE-PEGbiot and PE/PG/CL/DSPE-PEGbiot, respectively, at a 70/20/5/5 ratio (weight %). DSPE-PEGbiot allowed for biotin-mediated immobilization of liposomes onto streptavidin-coated biosensors (SA biosensors). When used for competition of chemokine binding or killing of bacteria, PE, PG, and CL liposomes consisted of 70% PC and 30% of the corresponding phospholipids. Phospholipids stored in chloroform were combined at the indicated ratios (for a total of 1 mg), and the solvent was evaporated using a SpeedVac concentrator (Thermo Fisher Scientific, Atlanta, GA). Dried lipid films were rehydrated for 1 hour at room temperature in 0.5 ml of phosphate-buffered saline (PBS), and large unilamellar liposomes were generated by extrusion (>11 passes) through a 0.1-μm pore-sized membrane using the Avanti Polar Lipids miniextruder. Liposomes were used immediately and prepared fresh for every experiment.

For calcein leakage assays, PE/PG/CL, PE/PG, and PE/PC liposomes were generated by extrusion as above by combining the indicated lipids at 70/25/5, 70/30, and 70/30 ratios (weight %), respectively. Dried lipid films were rehydrated for 1 hour at room temperature in 0.5 ml of 10 mM tris-HCl (pH 7.4) containing 70 mM calcein (Sigma-Aldrich, St. Louis, MO). Before extrusion, rehydrated phospholipids were subjected to five freeze-and-thaw cycles to ensure proper encapsulation of calcein. After extrusion, liposome-encapsulated calcein was purified and separated from any remaining free calcein by size exclusion using Sephadex 50 columns (Sigma-Aldrich) and calcein assay buffer [CAB; 10 mM tris-HCl (pH 7.4) and 85 mM NaCl] as elution buffer. Encapsulated calcein runs fast through the column forming an orange/yellow band, whereas free calcein lags as a bright-green band. Fractions (0.5 ml) were collected, and all orange fractions were pooled, stored at 4°C, and used within 4 days.

### Biolayer interferometry

Chemokine binding to full-length LPS and liposomes was analyzed by BLI using the Octet RED384 system (Pall ForteBio, Fremont, CA) as previously described (*22*). Before every run, SA biosensors (Pall ForteBio) were hydrated in PBS for 10 min. Then, sensors were equilibrated in PBS for 1 min, and biotinylated LPS (from *E. coli* O111:B4, InvivoGen, San Diego, CA) or liposomes containing DSPE-PEGbiot were immobilized to a final 1- to 3-nm response. Subsequently, sensors were washed in PBS for 1 min, followed by a 5-min incubation in PBS containing 0.05% bovine serum albumin. Baseline was stabilized in PBS for 5 min, then recombinant chemokine (500 nM in PBS) association was recorded for 500 s, and lastly, chemokine dissociation was monitored for 500 s by incubating the sensors with PBS alone. All steps were performed at 1000 rpm and 30°C. Background chemokine binding to SA biosensors uncoated or coated with PC liposomes consisting of PC/DSPE-PEGbiot (95:5 ratio) was analyzed in parallel and used as reference. Binding to these control sensors was subtracted from the binding recorded in sensors coated with LPS or every other liposome, respectively. Data were analyzed using the Octet Data Analysis software (Pall ForteBio).

### Chemokine binding to bacteria

The binding of AZ647-labeled chemokines was tested by FACS or confocal microscopy. For this, bacteria were grown in TSB to mid-early log phase (OD_600_ = 0.4 to 0.6) and washed once in AAB-85. Then, bacteria (1 × 10^+6^ CFU) were incubated with 0.3 μM fluorescent chemokine in 100 μl of AAB-85 at 37°C for 20 min. Where indicated, before the addition of the fluorescent chemokine, bacteria were preincubated with 0.3 μM unlabeled chemokines in 100 μl of AAB-85 at 37°C for 5 min. In addition, to test the effect of different lipids on the chemokine binding to bacteria, in some experiments, AZ647-labeled chemokines were preincubated with 100 μM PC liposomes containing 30% of PE, PG, or CL in 50 μl of AAB-85 at room temperature for 5 min. Then, 1 × 10^+6^ CFU of bacteria in 50 μl of AAB-85 were added to the liposome-chemokine mix and incubated at 37°C for 20 min. All bacterial samples were incubated with AZ647-labeled chemokines in microcentrifuge tubes. Then, samples were washed twice with 400 μl per sample of AAB-85, and bacteria were collected by centrifugation (9000*g* for 3 min).

For FACS analysis, washed bacteria were costained with 10 nM SYTO24 (Thermo Fisher Scientific) in 400 ml of AAB-85 in FACS tubes to distinguish the bacterial cells (SYTO24^+^) from debris (SYTO24^−^). Samples were analyzed in an LSRFortessa cytometer (BD Biosciences, Chicago, IL) by acquiring 30,000 events at 12 μl/min. Chemokine binding to SYTO24^+^ events was analyzed using FlowJo (BD Biosciences).

For confocal microscopy analysis, washed bacteria were fixed with 2% paraformaldehyde, immobilized on #1.5 coverslips of 0.17 ± 0.02 mm in thickness (Warner Instruments, Hamden, CT) previously coated with poly-d-lysine (0.1 mg/ml; Thermo Fisher Scientific) following the manufacturer’s recommendations and mounted using ProLong Diamond Antifade mountant with 4′,6-diamidino-2-phenylindole (DAPI) (Thermo Fisher Scientific) on superfrost plus microscope slides (Fisherbrand, Pittsburgh, PA). Samples were imaged with a confocal laser scanning microscope Zeiss LSM 880 or LSM980 (Carl Zeiss AG, Oberkochen, Germany). We used oil immersion alpha Plan-Apochromat 63×/1.4 Oil Corr M27 objective (Carl Zeiss) and Immersol 518F immersion medium [ne = 1.518 (30°C); Carl Zeiss]. A *z*-stack of images was collected across the entire cell. Identical image acquisition settings and optimal parameters for *x*, *y*, and *z* resolution were used in all samples from each independent experiment, and representative images for each condition in each experiment are shown with the same display range. Microscopy data processing, analysis, and quantification were done in ImageJ. To quantify bacterial binding, we measured the AZ647 fluorescence intensity of each bacterium at the *z* plane containing the highest signal, by generating a region of interest (ROI) around the cell using the oval tool. An equivalent ROI was generated at a region outside the bacterium, considered as background, and subtracted from the cell fluorescence intensity. The data were further analyzed and normalized against the control mean using GraphPad Prism 9.

### NAO and chemokine co-staining of *E. coli*

Localization of bacteria-bound CXCL9 was analyzed by Airyscan confocal microscopy in W3110 *E. coli* bacteria costained with NAO (Thermo Fisher Scientific). For this, bacterial cultures grown overnight were diluted 1:30 in TSB in the presence of 2 μM NAO and cultured in a laboratory shaker at 37°C and 220 rpm until OD_600_ ≥ 0.55. Then, bacteria were washed once with AAB-85, and 40 × 10^+6^ CFU were incubated with 0.3 μM CXCL9-AZ647 in 100 μl of AAB-85 at 37°C for 15 min. Bacteria were washed, fixed, immobilized onto poly-d-lysine–coated coverslips and mounted on microscope slides, as explained above. A *z*-stack of images was collected across the entire cell on an LSM980 confocal microscope equipped with Airyscan 2 detector (Carl Zeiss) using the super-resolution settings in frame mode and optimal parameters for *x*, *y*, and *z* resolution. NAO-524 was imaged with a 488-nm laser, a main beam splitter (MBS) 488/561, and a second beam splitter (SBS) SP 550, while NAO-630 was imaged with a 561-nm argon laser, an MBS 488/561, and an SBS LP 525. CXCL9-AZ647 was imaged with a 639-nm laser, an MBS 488/561/639, and an SBS LP 640. Airyscan postprocessing was performed using the standard parameters. A line was created along a bacterium, and the fluorescence profiles for each channel were generated using ImageJ and further normalized for the minimum and maximum fluorescence intensity of each independent channel.

### Thin-layer chromatography

Phospholipid composition of *E. coli* W3110 and BKT12 was analyzed by TLC as previously described (*33*). Total lipids were extracted from 100 ml of log-phase cultures by the acidic Blight Dyer method. For this, bacterial pellets were resuspended in 2 ml of 0.1 N of HCl and mixed with 5 ml of methanol and 2.5 ml of chloroform to generate a single-phase solution. After a 30-min incubation at room temperature, two phase solutions were created by adding 2.5 ml of 0.1 N of HCl and 2.5 ml of chloroform. Phases were separated by centrifugation (3000*g* for 25 min), and the organic lower phase was collected and evaporated using a SpeedVac concentrator (Thermo Fisher Scientific). The dried lipid film was resuspended by sonication in 100 μl of chloroform using a Bioruptor Pico (Diagenode, Denville, NJ). For TLC, samples were spotted on TLC Silica gel 60 plates (Millipore, Bedford, MA) with capillary tubes, and lipids were separated in a TLC developing chamber using a solution of chloroform/methanol/acetic acid (65:25:5, v/v) as mobile phase. Lipids were visualized by exposing the plates to iodine vapor by adding a few iodine crystals inside the chamber.

### OM permeabilization assay

The ability of chemokines to disrupt the OM of *E. coli* was studied by analyzing NPN permeability in the presence of chemokine or protamine. For this, mid-log phase cultures of W3110 and BKT12 *E. coli* strains were prepared in AAB-85 as detailed in the “Antimicrobial assays” section. Then, 50 μl per well of bacteria (2 × 10^+7^ CFU) in AAB-85 or 50 μl per well of buffer alone was added in black clear-bottom 96-well plates, and 25 μl of NPN (Sigma-Aldrich) diluted to 40 μM in AAB-85 was added to all wells. The baseline fluorescence signal (λ_ex_: 350 nm, λ_em_: 420 nm, no cutoff) was read at 37°C in a FlexStation 3 microplate reader (Molecular Devices, San Jose, CA) for ~25 min (one read every 2 min). Then, the read was interrupted, and 25 μl per well of buffer alone or PolB, protamine, CXCL8, or CXCL9 in AAB-85 (for a final protein concentration in the assay plate of 4 μM) was added in triplicates to the corresponding wells before resuming the read in the microplate reader for an additional 75 min. The average background NPN fluorescence signal recorded at each time point in wells with buffer alone (no bacteria) was subtracted from all samples, and the response curves were adjusted to zero subtracting the first read from all the values recorded over time in each well. The average maximum fluorescence read (100% OM permeabilization) in PolB-treated bacteria was determined for each *E. coli* strain and used as reference to calculate the percentage of OM permeabilization at each time point for each treatment and strain.

### Time-to-kill assays

The kinetics of chemokine effects on bacterial growth and bacterial killing were analyzed by FACS. For this, 125 μl per well of a bacterial suspension at 10^+6^ CFU/ml in AAB-85 was added in a U-bottom 96-well plate in the presence of buffer alone or the indicated concentrations of chemokine, hBD3, or protamine. Then, plates were incubated at 37°C for 3 hours and 25-μl aliquots of each sample were collected at 20, 60, 120, and 180 min for analysis. Aliquots of the inputs before incubation were also collected for determination of the initial number of bacteria (time 0). These 25-μl aliquots were stained in 400 μl of AAB-85 containing 10 nM SYTO24 and 1.25 μM SYTOX-Orange (both from Thermo Fisher Scientific) in FACS tubes. SYTOX-Orange only permeates dead bacteria with compromised plasma membrane integrity, whereas SYTO24 stains both live and dead bacteria, allowing us to also distinguish bacteria (SYTO24^+^) from debris (SYTO24^−^) during analysis. FACS tubes were incubated 5 min at room temperature, and events were acquired for 30 s at 12 μl/min in an LSRFortessa cytometer (BD Biosciences). Data were analyzed using FlowJo (BD Biosciences).

### Calcein leakage assay

The ability of chemokines and other AMPs to lyse phospholipid membranes was studied by liposome calcein leakage assays. The fluorescent dye calcein is self-quenched at high concentrations inside liposomes, but upon lysis of the liposome and release into the extraliposomal buffer, calcein regains its fluorescence properties. PE/PG/CL, PE/PG, and PE/PC liposomes encapsulating calcein were prepared and purified in CAB as detailed above (see the “Liposomes” section). To determine the appropriate volume of liposomes for these assays, calcein release in 10-fold serial dilutions of liposome samples after incubation with CAB containing 0.5% Triton X-100 was first titrated using a FlexStation 3 microplate reader (Molecular Devices). Liposome sample volumes causing a 5- to 10-fold increase in the calcein fluorescent signal (λ_ex_: 485 nm, λ_em_: 515 nm) over baseline were selected for each liposome preparation. Liposomes were first diluted in CAB, and 50 μl per well was added in clear-bottom black 96-well plates (Greiner, Monroe, NC). Baseline fluorescence was read for 5 to 10 min in the kinetic mode of the FlexStation 3 reader taking reads every 15 to 20 s. Then, the read was interrupted, the plate was taken back to the bench, and 25 μl per well of buffer alone, chemokine, or protamine prepared in CAB at three times the desired final concentration was added. The plate was returned to the microplate reader, and the read resumed appending the additional read points to the baseline reads. Last, ~30 min later, the read was interrupted again to add 25 μl per well of CAB containing 0.5% Triton X-100, and the fluorescence signal was recorded for additional 5 min to obtain a measurement of the maximum calcein release for each sample. Fluorescence signals were normalized to baseline, and the percentage of calcein release for each sample was calculated as the percentage fluorescence relative to the maximum fluorescence signal obtained after Triton X-100 addition. The percentage of released calcein observed for each type of liposome after treatment with buffer alone was subtracted from the corresponding liposome samples treated with the different chemokines or protamine.

### SAXS experiments with model membranes

Methods used for SAXS experiments and data fitting were based around those that have been previously described (*36*, *75*). Liposomes were prepared for SAXS experiments as previously described (*36*, *76*). Briefly, lyophilized phospholipids DOPG and DOPE purchased from Avanti Polar Lipids were dissolved in chloroform stocks at 20 mg/ml. Model membrane lipid compositions were prepared from the lipid stock solutions at a PG/PE 20/80 molar ratio. The lipid composition was evaporated under nitrogen and then desiccated overnight under vacuum to form a dry lipid film, which was resuspended in aqueous 140 mM NaCl and 10 mM Hepes (pH 7.4) to a concentration of 20 mg/ml. Lipid suspensions were incubated overnight at 37°C, sonicated until clear, and then extruded through a 0.2-μm pore-sized Anopore membrane filter (Whatman) to form small unilamellar vesicles (SUVs). Lyophilized CCL5, CCL19, and CXCL11 powder were solubilized in aqueous 140 mM NaCl and 10 mM Hepes (pH 7.4) and incubated with SUVs at peptide-to-lipid (P/L) charge ratios of 1/6, 1/4, 1/2, 1/1, or 3/2. Samples were hermetically sealed into quartz capillaries (Hilgenberg GmbH, Mark-tubes) for measurements taken at the Stanford Synchrotron Radiation Lightsource (beamline 4-2) using monochromatic x-rays with an energy of 9 keV.

The scattered radiation was collected using a DECTRIS PILATUS3 X 1M detector (pixel size, 172 μm), and the resulting 2D SAXS powder patterns were integrated using the Nika 1.50 (*77*) package for Igor Pro 6.31 and FIT2D (*78*). Using OriginLab software, the integrated scattering intensity *I*(*q*) was plotted against *q*. Ratios of the measured peak positions were compared with those of permitted reflections for different crystal phases to identify the phase(s) present in each sample. A linear regression through points corresponding to the peaks was used to calculate the lattice parameter, *a*, of each identified cubic phase. For a cubic phase, each peak is represented by a point with coordinates of the assigned reflection (in terms of Miller indices *h*, *k*, and *l*) and *q*. For a cubic phase, *q* = (2π/*a*)√(*h*^2^ + *k*^2^ + *l*^2^). Therefore, the slope of the regression (*m* = 2π/*a*) of *q* versus √(*h*^2^ + *k*^2^ + *l*^2^) can be used to estimate *a*. The mean NGC was estimated as *<k>* = $\frac{2\pi \chi }{A_{0}a^{2}}$ , where *A*_0_ and χ are the dimensionless surface area per unit cell and Euler-Poincaré characteristic, respectively, for each cubic phase. For *Pm*3*m*, *A*_0_ = 1.919 and χ = −2; for *Im*3*m*, *A*_0_ = 2.345 and χ = −4; and for *Ia*3*d*, *A*_0_ = 3.091 and χ = −8. For spectra with coexisting *Pm*3*m* and *Im*3*m* cubic phases, the ratio of their lattice parameters was noted to satisfy the Bonnet ratio of 1.279.

### Expression and purification of recombinant CCL20-His

Recombinant CCL20-His was expressed and purified using a protocol adapted from previously published methods (*79*). pNAN plasmids containing the human *CCL20* coding sequence in frame with a C-terminal short linker (Ser-Gly-Gly-Ser) and a 6×His tag, as well as an Amp resistance gene, were purchased from GenScript (Piscataway, NJ). Plasmid (25 ng) was transformed into 50 μl of competent Rosetta BL21(DE3) pLysS cells via the heat shock method. Cells were streaked onto Amp agar plates (150 μg/ml) and incubated at 37°C overnight. A single colony was selected for inoculation and grown overnight in 2× yeast extract tryptone broth (2×YT) with Amp (150 μg/ml). For each liter of 2×YT broth with Amp (150 μg/ml), 20 ml of inoculum was added and grown to OD_600_ = 0.6 at 37°C and induced with 0.5 mM isopropyl-β-d-thiogalactopyranoside for 6 hours before harvesting at 4000*g* and storing overnight at −20°C. For each liter of broth, cells were resuspended in 40 ml of lysis buffer [50 mM tris-HCl (pH 8.0), 300 mM NaCl, benzonase (0.1 mg/ml), 10 mM MgCl_2_, and one tablet of cOmplete protease inhibitor cocktail (Millipore, Bedford, MA)] and lysed on ice using a Branson 102-C sonifier at 70% power for 3 min (5-s on and 25-s off). After centrifuging at 12,000*g* to clarify, the maroon-colored inclusion body pellet was resuspended in 10 ml of buffer AD [6 M guanidine HCl, 50 mM tris, and 1 mM tris(2-carboxyethyl)phosphine (pH 8.0)] and heated for 30 min in a 60°C water bath while passing the suspension through 16- and 20-gauge needles to break up the pellet and dissolve the inclusion bodies. This solution was centrifuged at 24°C for 30 min at 12,000*g* to pellet any further insoluble membranes and cellular contents. If centrifugation was insufficient to clarify supernatant, then the solution was filtered at 0.45 μm to achieve an optically clear solution.

For each liter of broth used, 3 ml of Ni Sepharose Excel resin was used. Resin was washed with 5 column volumes (CV) of Milli-Q water and then equilibrated with 5 CV of buffer AD. The solubilized inclusion body solution was added to resin and allowed to drip by gravity flow, followed by a 10-CV wash with buffer AD. CCL20-His bound to the column was eluted using 2-CV buffer BD [6 M guanidine HCl and 100 mM NaOAc (pH 4.5)] twice. Before progressing, the protein was diluted to 1 mg/ml in buffer BD, quantitated by estimation via its absorbance at 280 nm, and diluted dropwise into 2× volume of cystine/cysteine refolding solution [6.5 mM cysteine, 0.65 mM cystine, and 300 mM NaHCO_3_ (pH 7.4)] and allowed to stir overnight at room temperature. To finalize refolding, the protein solution was dialyzed against 600× volumes of PBS overnight at 4°C to bring the final guanidine HCl concentration to submillimolar concentrations. Refolded CCL20-His was clarified by centrifugation at 12,000*g* for 45 min, followed by 0.22-μm filtration. Sample was concentrated using 10-kDa Amicon ULTRA concentrators (Millipore) until an appropriate concentration was reached. Residual guanidine was removed by concentrating and diluting three times with 5× volumes of PBS.

### MIC assay

MICs for chemokines and antibiotics were determined in MHB (Sigma-Aldrich). For this, twofold serial dilutions (90 μl per well) of CCL20-His, Tet, and Amp were prepared in U-bottom 96-well plates. *E. coli* W3110 strain was grown to mid-early log phase (OD_600_ = 0.4 to 0.6), and 50,000 CFU of bacteria in 10 μl of MHB were added to each well (final volume = 100 μl per well; final bacteria concentration = 5 × 10^+5^ CFU/ml). Plates were incubated at 37°C for 18 hours, and MIC was determined as the lower concentration of chemokine/antibiotic showing no evidence of bacterial growth analyzed by luminometry using the BacTiter-Glo Microbial Cell Viability Assay (Promega).

### Microbial resistance induction assay

To determine the ability of bacteria to develop resistance against antimicrobial chemokines, we monitored over the course of 14 days the change in MIC caused by the exposure of bacteria to a sublethal dose of each antimicrobial agent. For this, *E. coli* W3110 strain was grown in MHB in the presence of CCL20-His, Amp, or Tet at a concentration equivalent to 0.5 × MIC. The initial MIC (20 μM CCL20, 14.3 μM Amp, and 1.1 μM Tet) was determined in duplicate by MIC assay. The first day, 50,000 CFU per well of bacteria were cultured in 100 μl of MHB supplemented with the corresponding dose of chemokine/antibiotic in a U-bottom 96-well plate. Two independent bacterial cultures were initiated per treatment and analyzed individually. Bacteria were subcultured every 18 hours in 100 μl per well of MHB containing a fresh dose of chemokine/antibiotic. On selected days, MIC was determined for each treatment as explained above, and chemokine/antibiotic dose was adjusted to the new 0.5 × MIC.

### Statistical analysis

Data were analyzed using GraphPad Prism 9. Statistical tests applied for the analysis of each dataset are detailed in the corresponding figure legend.
