# Supplementary material for: Chemokines kill bacteria without triggering antimicrobial resistance by binding anionic phospholipids
Source: Sci Adv. 2025 Jun 6;11(23):eads2675. doi: 10.1126/sciadv.ads2675 (PMC12143392; doi:10.1126/sciadv.ads2675)
Supplement: Supplementary file 1 — Figs. S1 to S7 Table S1 [file sciadv.ads2675_sm.pdf]

Science Advances
AAAS

Supplementary Materials for

Chemokines kill bacteria without triggering antimicrobial resistance by binding anionic phospholipids

Sergio M. Pontejo *et al.*

Corresponding author: Sergio M. Pontejo, jesussergio.martinpontejo@nih.gov

Sci. Adv. **11**, eads2675 (2025)
DOI: 10.1126/sciadv.ads2675

This PDF file includes:

Figs. S1 to S7
Table S1

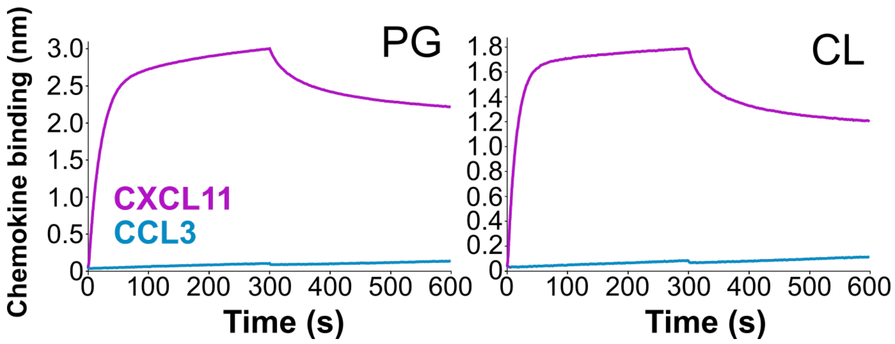

Figure S1. CCL3 does not bind phosphatidylglycerol or cardiolipin.
Binding of CCL3 (500 nM) to phosphatidylcholine liposomes containing 30% PG or CL, as indicated on the top right corner of each graph, was analyzed by BLI. CXCL11 was used as positive control. PG, phosphatidylglycerol; CL, cardiolipin.

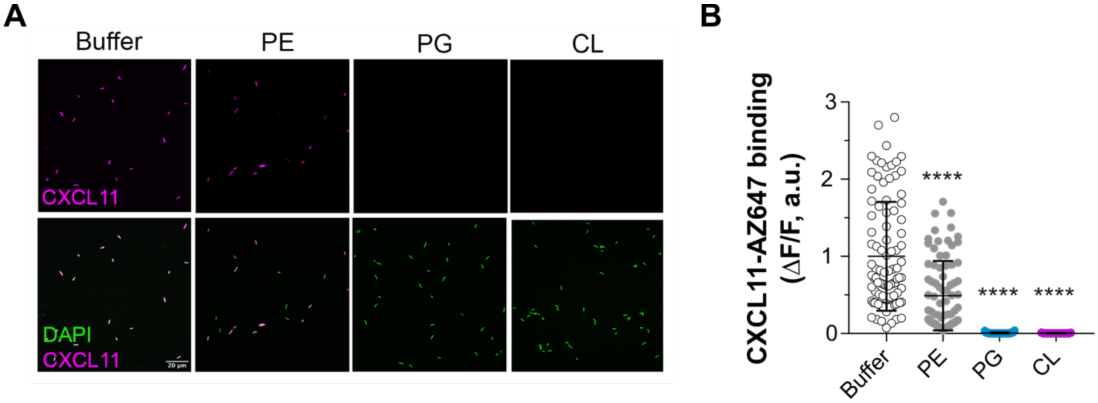

Figure S2. Liposomes containing PG or CL block CXCL11 binding to bacteria.
(**A**) Representative images of the binding of CXCL11-AZ647 (0.3 μM) to bacteria in the presence of buffer or PC liposomes (100 μM) containing 30% of PE, PG, or CL, as indicated above each image column. Top row, staining of CXCL11-AZ647 alone; Bottom row, merge of CXCL11-AZ647 staining with DAPI. A white scale bar (20 μm) is inserted in the bottom left image. **(B)** Quantification of the fluorescence intensity of the binding of CXCL11-AZ647 per bacterium in each treatment group. Each dot represents one bacterium (n ≈ 100). Data are shown as mean ± SD from one experiment representative of 2 independent experiments and were compared to the buffer-treated group using one-way ANOVA with Tukey test for multiple comparisons (****, $p<0.0001$). DAPI, 4′,6-diamidino-2-phenylindole; CL, cardiolipin; PG, phosphatidylglycerol; PE, phosphatidylethanolamine.

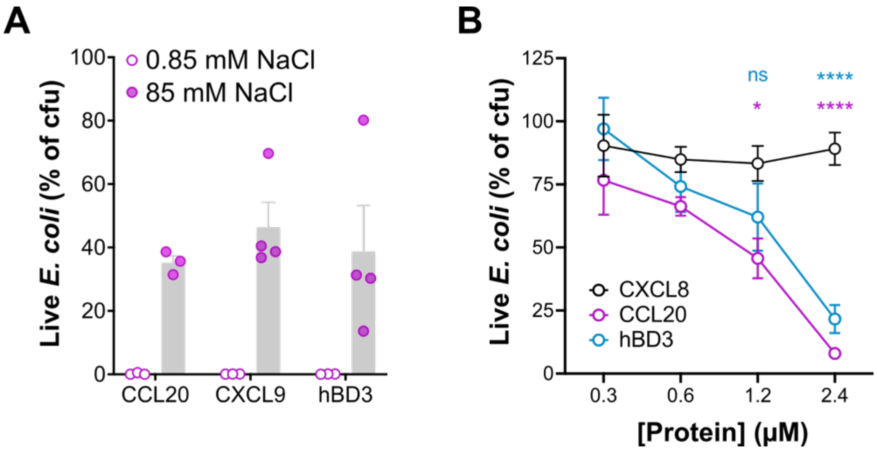

Figure S3. Chemokines and defensins exert reduced but significant antimicrobial activity in 85 mM NaCl buffer. (A) High salt concentrations reduce antimicrobial activity by chemokines. Antimicrobial assay showing the number of surviving cfu after incubation (2 h, 37° C) of *E. coli* (W3110 strain) bacteria (10^{+5} cfu) with CCL20, CXCL9 or hBD3 (1.2 µM) in assay buffer containing 0.85 mM (open dots) or 85 mM (filled dots) NaCl. Data are represented as mean ± SEM % of cfu (gray bars) relative to the number of cfu counted in samples treated with buffer alone. Data are from 3-4 independent experiments (dots) combined with biological triplicates in each experiment. **(B)** Chemokines are as potent antimicrobials as hBD3 in the presence of 85 mM NaCl. *E. coli* (10^{+5} cfu) survival after incubation (2 h, 37° C) with increasing doses of CCL20, hBD3 or CXCL8 (non-antimicrobial control) in assay buffer containing 85 mM NaCl. Data were analyzed as in A and correspond to 3 independent experiments combined, with biological triplicates in each experiment. Statistical differences (color-coded above each concentration data point) with the CXCL8 control group were analyzed by two-way ANOVA with Bonferroni test for multiple comparisons. ns, not significant; *, $p < 0.05$; ****, $p < 0.0001$. hBD3, human beta-defensin 3; cfu, colony forming units; NaCl, sodium chloride.

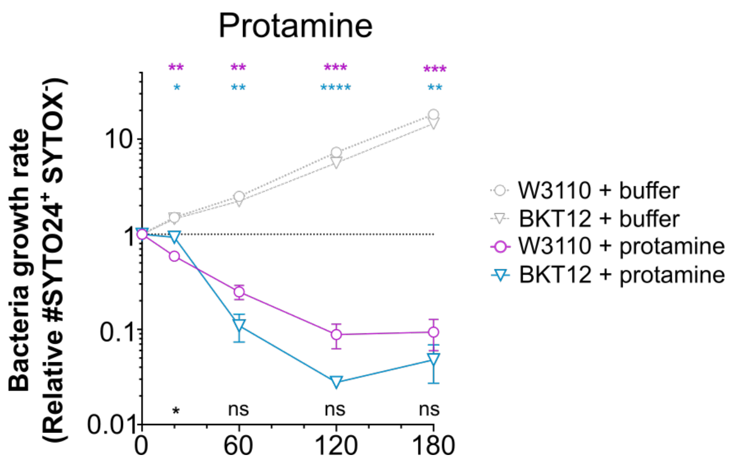

Figure S4. Protamine is a potent bactericidal peptide. Time-to-kill assay showing the number of live W3110 or BKT12 bacteria over time relative to the initial bacterial input (time 0) after incubation with buffer alone or 1.2 μM of protamine (as indicated in the legend). Live bacteria were quantified by co-staining with SYTO24 (stains dead and live bacteria) and SYTOX (stains only dead bacteria) using FACS. Horizontal dotted line indicates the ratio = 1 (no growth). Data are summarized as the mean ± SEM of 4 independent experiments combined, with biological triplicates in each experiment. Data were log-transformed and analyzed by two-way ANOVA with Tukey test for multiple comparisons. Results of statistical analyses for the comparison of buffer vs protamine for each bacterial strain are color-coded and indicated above each graph. Results of statistical analyses for the comparison of protamine-treated W3110 vs BKT12 are indicated above the x axis. ns, not significant; *, $p < 0.05$; **, $p < 0.01$; ***, $p < 0.001$; ****, $p < 0.0001$.

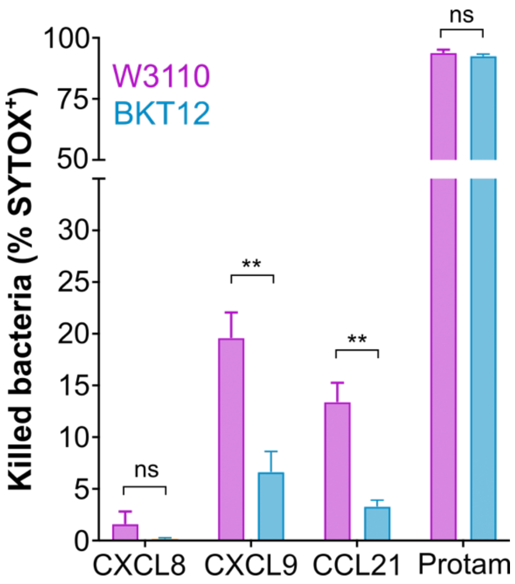

Figure S5. CL-deficient bacteria are resistant to direct killing by antimicrobial chemokines. Quantification of the % of dead W3110 and CL-deficient BKT12 bacteria (as indicated in the inset) 90 min after treatment with 4.8 µM of CXCL8, CXCL9, CCL21 or protamine. Dead bacteria were quantified by FACS as the % of $SYTOX^+$ cells relative to the total number of $SYTO24^+$ bacteria. Data are the mean ± SD of 3 biological replicates from one experiment representative of 3 independent experiments. Statistical differences (W3110 vs BKT12) were analyzed by multiple unpaired 2-tail t test. ns, not significant; **, $p < 0.01$. Protam, protamine.

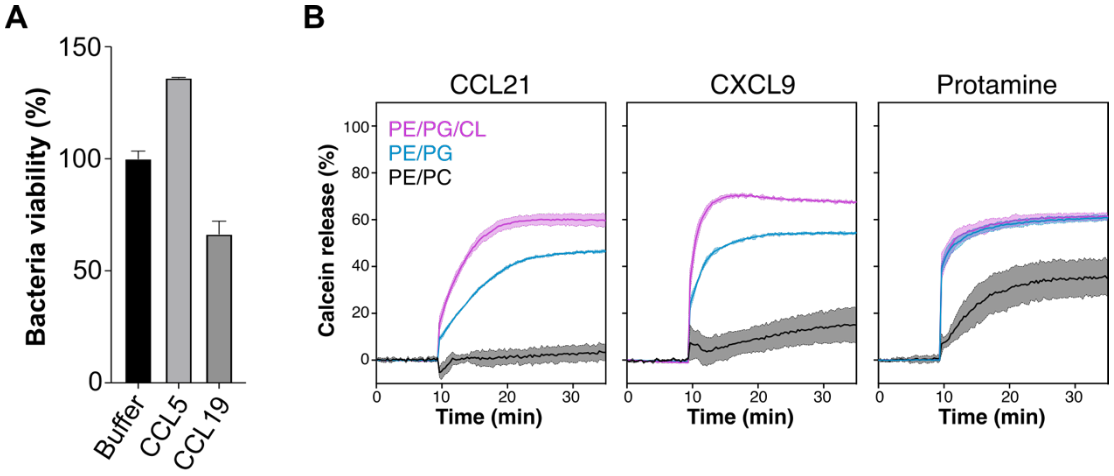

Figure S6. CCL5 is not antimicrobial and CL promotes liposomal membrane disruption by low doses of antimicrobial chemokines. (A) *E. coli* (W3110 strain) bacterial viability determined using the BacTiter-Glo kit after incubation (2 h, 37°C) of 10^{+5} cfu with buffer alone or 1.2 µM of CCL5 or CCL19. Bars represent the mean ± SD of biological triplicates from one experiment representative of 2 independent experiments. **b)** Calcein-leakage assays showing the % of calcein released by 0.15 µM of CCL21, CXCL9 or protamine (as indicated above each graph) from PE/PG/CL, PE/PG or PE/PC liposomes (CCL21 graph inset) relative to the maximum calcein release observed when liposomes were incubated with TritonX-100. Solid lines represent the mean of 3 biological replicates. Colored shaded area around the lines represents the SD. Data are from one experiment representative of 3 independent experiments. cfu, colony forming units; CL, cardiolipin; PG, phosphatidylglycerol; PE, phosphatidylethanolamine; PC, phosphatidylcholine.

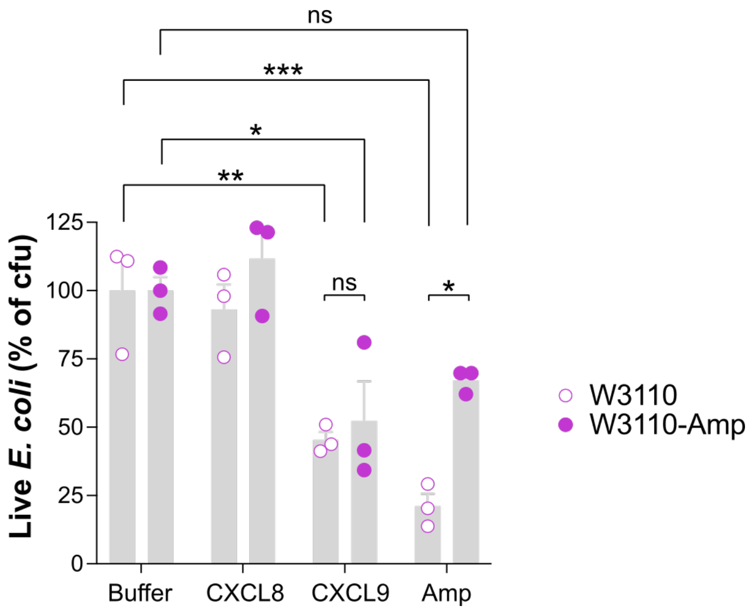

Figure S7. CXCL9 kills ampicillin-resistant *E. coli*.
Parental W3110 and ampicillin-resistant W3110-Amp (as indicated in the legend) *E.coli* strains were incubated with buffer, 1.2 µM of CXCL8 (non-antimicrobial) or CXCL9 (antimicrobial), or 5 µg/ml of ampicillin (Amp) for 2h at 37°C. Bacterial viability was determined by cfu assays. Bars represent the mean ± SEM % of surviving cfu of biological triplicates relative to the "Buffer" group for each bacterial strain. Data are from one experiment representative of 2 independent experiments and were analyzed by two-way ANOVA with Tukey's test for multiple comparisons (ns, not significant; *, $p < 0.05$; **, $p < 0.01$; ***, $p < 0.001$). cfu, colony forming units.

Table S1. Biochemical properties of the human chemokines and AMPs used in this study. MW, molecular weight; pI, isoelectric point; R/K content, percentage of arginine and lysine residues in the primary amino acid sequence.

	Antimicrobial activity	Lipid binding	MW (kDa)	pI	R/K content (%)
CCL3	NO	NO	7.8	4.8	8.6
CCL5	NO	NO	7.8	9.2	14.8
CCL11	YES	YES	8.3	9.9	21.6
CCL19	YES	YES	8.8	9.8	16.9
CCL20	YES	YES	8.0	9.7	17.2
CCL21	YES	YES	12.2	12.3	22.5
CCL22	NO	NO	8.0	9.1	16.4
CCL23	NO	NO	11.3	9.2	17.2
CXCL5	NO	NO	8.0	8.8	13.5
CXCL8	NO	NO	8.4	9.0	19.4
CXCL9	YES	YES	11.7	10.3	26.3
CXCL11	YES	YES	8.3	10.0	23.2
hBD3	YES	n.a.	5.2	10.1	28.9
Protamine	YES	n.a.	4.5	13.3	63.6
